# TBK1 and GABARAP family members suppress Coxsackievirus B infection by limiting viral production and promoting autophagic degradation of viral extracellular vesicles

**DOI:** 10.1371/journal.ppat.1010350

**Published:** 2022-08-31

**Authors:** Savannah Sawaged, Thomas Mota, Honit Piplani, Reetu Thakur, Deepti Lall, Elizabeth McCabe, Soojung Seo, Fayyaz S. Sutterwala, Ralph Feuer, Roberta A. Gottlieb, Jon Sin

**Affiliations:** 1 The Smidt Heart Institute, Cedars-Sinai Medical Center, Los Angeles, California, United States of America; 2 The Center for Neural Science and Medicine, Regenerative Medicine Institute, Cedars-Sinai Medical Center, Los Angeles, California, United States of America; 3 Department of Biological Sciences, University of Alabama, Tuscaloosa, Alabama, United States of America; 4 Department of Medicine, Women’s Guild Lung Institute, Cedars-Sinai Medical Center, Los Angeles, California, United States of America; 5 The Integrated Regenerative Research Institute at San Diego State University, San Diego, California, United States of America; University of Maryland, UNITED STATES

## Abstract

Host-pathogen dynamics are constantly at play during enteroviral infection. Coxsackievirus B (CVB) is a common juvenile enterovirus that infects multiple organs and drives inflammatory diseases including acute pancreatitis and myocarditis. Much like other enteroviruses, CVB is capable of manipulating host machinery to hijack and subvert autophagy for its benefit. We have previously reported that CVB triggers the release of infectious extracellular vesicles (EVs) which originate from autophagosomes. These EVs facilitate efficient dissemination of infectious virus. Here, we report that TBK1 (Tank-binding kinase 1) suppresses release of CVB-induced EVs. TBK1 is a multimeric kinase that directly activates autophagy adaptors for efficient cargo recruitment and induces type-1 interferons during viral-mediated STING recruitment. Positioning itself at the nexus of pathogen elimination, we hypothesized that loss of TBK1 could exacerbate CVB infection due to its specific role in autophagosome trafficking. Here we report that infection with CVB during genetic *TBK1* knockdown significantly increases viral load and potentiates the bulk release of viral EVs. Similarly, suppressing *TBK1* with small interfering RNA (siRNA) caused a marked increase in intracellular virus and EV release, while treatment *in vivo* with the TBK1-inhibitor Amlexanox exacerbated viral pancreatitis and EV spread. We further demonstrated that viral EV release is mediated by the autophagy modifier proteins GABARAPL1 and GABARAPL2 which facilitate autophagic flux. We observe that CVB infection stimulates autophagy and increases the release of GABARAPL1/2-positive EVs. We conclude that TBK1 plays additional antiviral roles by inducing autophagic flux during CVB infection independent of interferon signaling, and the loss of TBK1 better allows CVB-laden autophagosomes to circumvent lysosomal degradation, increasing the release of virus-laden EVs. This discovery sheds new light on the mechanisms involved in viral spread and EV propagation during acute enteroviral infection and highlights novel intracellular trafficking protein targets for antiviral therapy.

## Author summary

Coxsackievirus B (CVB) is a significant human enterovirus that can cause myocarditis, meningitis, and pancreatitis. The subversion of host immunity and mechanisms of viral dissemination are critical factors which promote pathogenesis. We had previously reported that following infection, CVB becomes engulfed by autophagosomes which evade lysosomal degradation and instead get released as infectious extracellular vesicles (EVs). In this current study, we report that in addition to its traditional role in interferon-mediated antiviral signaling, TANK-binding kinase (TBK1) is crucial in limiting viral production and EV-based viral egress through the autophagy pathway. Indeed, in the absence of TBK1, we observe (i) a disruption in autophagic flux, (ii) significant increases in intracellular viral burden and viral EV release, and (iii) elevated viral load in both *in vitro* and *in vivo* models of infection. EVs isolated from TBK1-deficient cells or mice treated with the TBK1-inhibitor Amlexanox were more infectious compared to controls. In all, the dual role TBK1 plays in suppressing viral escape in addition to mediating antiviral immunity makes it a promising therapeutic target for the treatment of CVB infection.

## Introduction

Viruses require host cells for survival and replication. Coxsackievirus B (CVB) is a common enterovirus in the *Picornaviridae* family that exhibits multiorgan tropism [1]. CVB is known to cause autoinflammatory diseases such as myocarditis, meningitis, and pancreatitis [2–4]. It is also linked to multiple neurological pathologies including amyotrophic lateral sclerosis (ALS) and frontotemporal dementia (FTD) [5,6]. CVB is composed of four structural capsid proteins and seven non-structural proteins which are responsible for viral replication and host cell manipulation [7–9]. CVB activates host cell autophagy as a mode of viral spread via the release virus-laden extracellular vesicles (EVs) [10–12]. Our group observed that autophagic flux is blocked during later stages of infection when EVs are shed [10]. We saw that EVs released during CVB infection were enriched with LC3-II, suggesting their origin from autophagosomes. Other groups have also documented the ability for CVB to facilitate efficient infection through *en bloc* transmission of the virus via EVs [13]. MicroRNAs and protein aggregates are co-secreted with viral particles in EVs and may also facilitate viral infection [14–16]. Though EV-mediated viral spread has been described for some time now, the molecular mechanisms that contribute to EV release are poorly understood.

TANK-binding kinase 1 (TBK1) is a serine-threonine kinase which belongs to the Iκκ family of kinases that play central roles in immunity and autophagy. TBK1 was initially described for its ability to stimulate NFκB [17]. Upon activation, TBK1 directly phosphorylates interferon regulatory factor 3 (IRF3) to induce its translocation to the nucleus and transcription of antiviral cytokines. Though TBK1’s major role as a protein kinase has classically been studied in the context of cellular innate immunity, more recent research highlights TBK1’s expanded role as a significant kinase in autophagy for pathogen clearance [18,19]. Defined as a highly conserved, catabolic process, autophagy maintains cellular homeostasis by directing damaged organelles, protein aggregates, and pathogens to the lysosome for degradation [20]. Autophagy is initiated by *de novo* formation of the double membrane phagophore by autophagy-related protein 5 (ATG5) and ATG12 [21,22]. During the elongation of the phagophore, autophagy adaptor proteins optineurin and p62/SQSTM1 are phosphorylated by TBK1 for efficient target selection and sequestration to the forming autophagosome [18,23]. ATG8 family members, including LC3, GABARAPL1, and GABARAPL2, are simultaneously phosphorylated by TBK1 to facilitate closure of the autophagosome and autophagosome fusion with the lysosome. Though TBK1 is not required for the initiation of autophagy, recent findings report its relevance in premature removal of ATG8 family members from autophagosomes, thereby disrupting fusion with the lysosome [24]. Through direct phosphorylation of the ATG8 family members LC3C and GABARAPL2, TBK1 prevents the ATG4 protease from prematurely cleaving the glycine-phosphatidylethanolamine bond and releasing them from the autophagosome.

Given the importance of TBK1 in promoting autophagosome closure and fusion with the lysosome, we asked whether TBK1 plays a role in attenuating CVB-induced autophagy and viral extracellular release of EVs. It has been reported that CVB does not induce significant type I interferon responses [7], therefore we focused our studies on the effects of TBK1 on autophagic signaling. We found that genetic knockout or silencing of TBK1 increased intracellular viral content and promoted efficient release of extracellular virus. EVs isolated from *TBK1*-silenced cells not only contained more viral capsid protein VP1 but also more autophagosomal lipidated LC3 and GABARAP family members. Additionally, these EVs showed increased viral infection efficacy as revealed by plaque assay. Consistent with these findings, direct activation of TBK1 by Manassantin B (ManB) significantly reduced viral infection and viral dissemination via EVs [25]. We hypothesize that in addition to its canonical role in antiviral cytokine activation, TBK1 reduces viral production by increasing delivery of virus-laden autophagosomes to lysosomes for degradation.

## Results

### Germline knockout or silencing TBK1 increases viral infection and release

**Fig 1:**
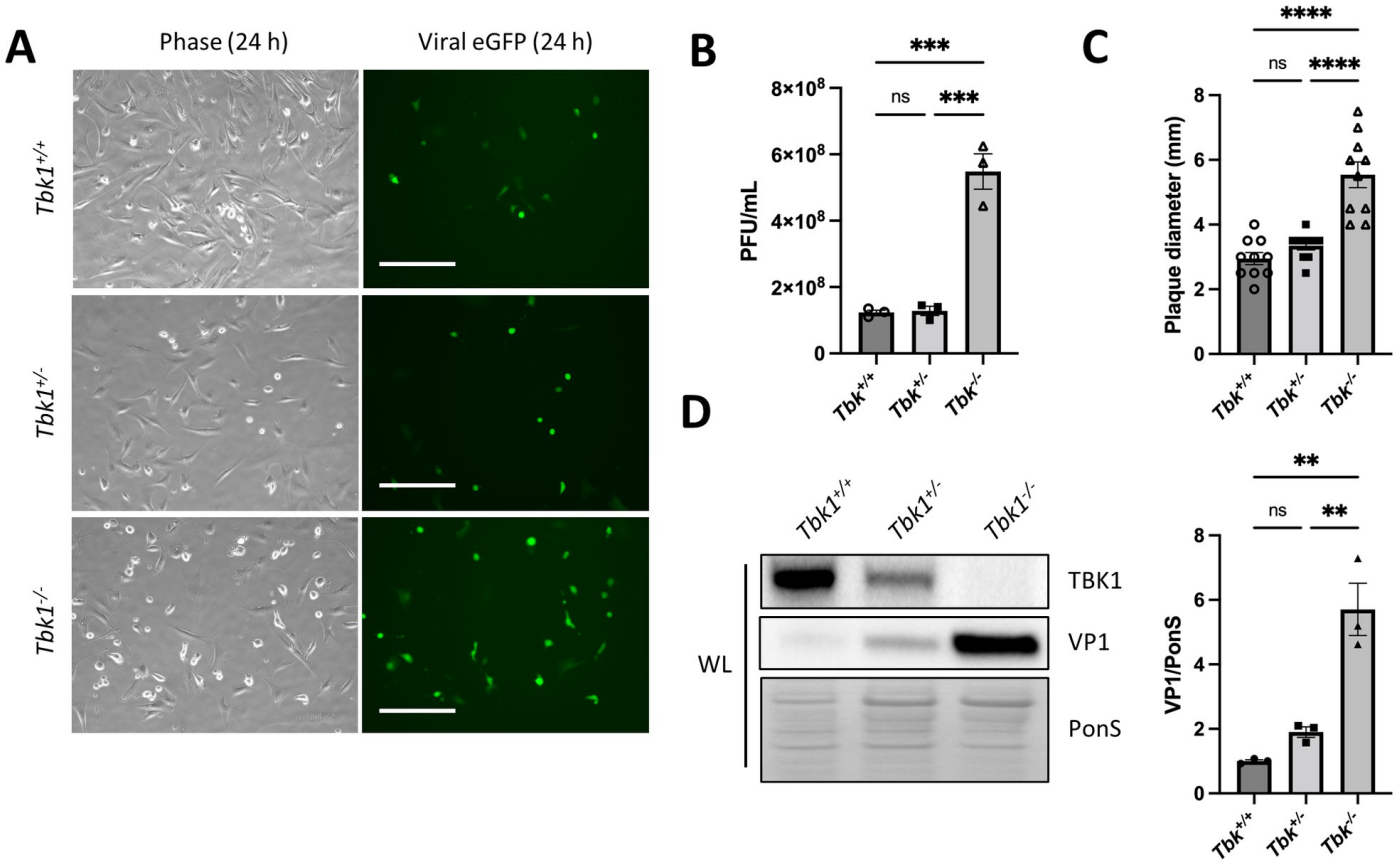
CVB infection increases in a genetic knockout of *TBK1*. Wild-type (*TBK1*^*+/+*^), heterozygous knockout (*TBK1*^*+/-*^), and homozygous knockout (*TBK1*^*-/-*^), mouse embryonic fibroblasts (MEFs) were infected with eGFP-expressing coxsackievirus B at a multiplicity of infection of 10 (MOI 10). (A) Fluorescence microscopy of infected MEFs at 24 h post-infection (p.i.). Phase contrast images show similar cell density at 24 h p.i. Scale bars represent 100 μm. (B) Extracellular viral titers of infected cells at 24 h as measured by plaque assay. ***, *p* < 0.001. Student *t* test n = 3. (C) Diameter of viral plaques. ****, *p* < 0.0001, Student *t* test; n = 10. (D) Western blots of infected cells at 24 h p.i. including densitometry. A representative Western blot is shown. **, *p* < 0.01, Student *t* test; n = 3. Data are representative of 3 experiments. WL = whole lysate.

To investigate whether TBK1 modulation influences viral infection, we utilized mouse embryonic fibroblasts (MEFs) from wildtype (*TBK1*^*+/+*^), heterozygous *TBK1* knockout (*TBK1*^*+/-*^), and homozygous *TBK1* knockout (*TBK1*^*-/-*^) mice. The use of MEFs was necessary since homozygous *TBK1* deletion is embryonic lethal [26]. MEFs from all three mouse lines were infected with enhanced green fluorescent protein (eGFP)-expressing CVB (eGFP-CVB) at a multiplicity of infection (MOI) of 10 (MOI 10). We found that at 24 hours (h) post-infection (p.i.), *TBK1*^*-/-*^ MEFs displayed increased viral infection compared to *TBK1*^*+/+*^ and *TBK1*^*+/-*^ MEFs, as evidenced by eGFP-expressing cells (10% increase; *, *p* < 0.05) (Fig 1A). Plaque assays on the media from these cells showed statistically significant increases in extracellular viral release and plaque diameter in *TBK1*^*-/-*^ MEFs at 24 h p.i. compared to both *TBK1*^*+/+*^ and *TBK1*^*+/-*^ MEFs (Fig 1B and 1C). We hypothesize that many of these larger plaques could be attributable to an increase in the presence of CVB-laden EVs. Previous literature demonstrated that these types of enterovirus-induced vesicles (including coxsackievirus-induced vesicles) could contain many infectious virions in a single vesicular structure [13]. Thus, plaques formed from infection of a single EV could be formed by numerous virions, potentially increasing viral burden more quickly and accelerating radial viral spread.

Western blots on cell lysates revealed significantly increased levels of viral capsid protein VP1 in *TBK1*^*-/-*^ (Fig 1D). To understand whether the increase in viral release in *TBK1*^*-/-*^ cells was due to augmented intracellular viral replication, we performed plaque assays on cell lysates and supernatants from *TBK1*^*+/+*^ and *TBK1*^*-/-*^ MEFs (S1 Fig). Outstandingly, both intracellular and extracellular *TBK1*^*-/-*^ viral titers were significantly increased compared to *TBK1*^*+/+*^ viral titers, revealing that genetic loss of *TBK1* impacts both intracellular viral content and extracellular viral release.

**Fig 2:**
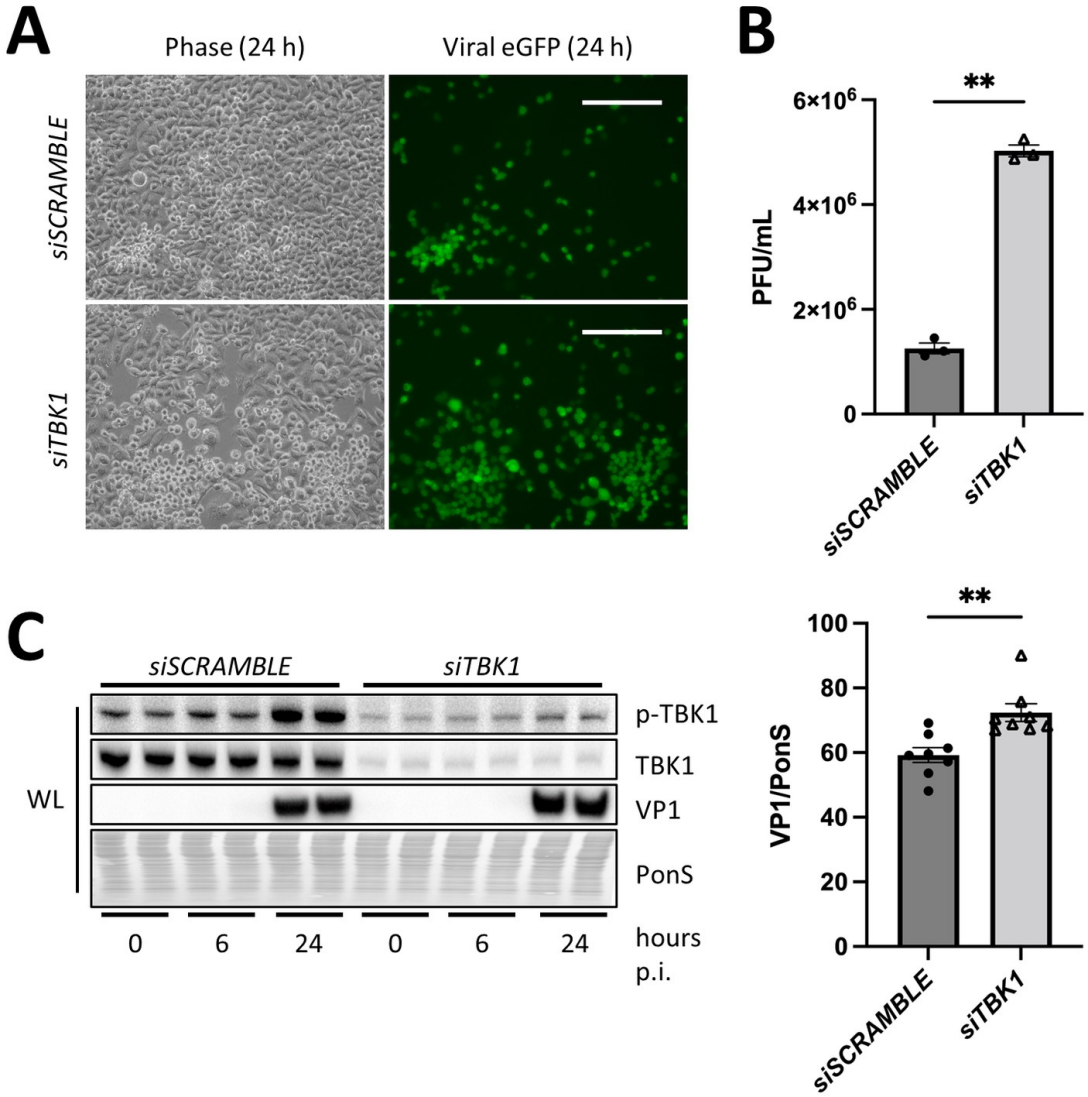
Silencing *TBK1* increases CVB infection. HeLa cells were treated with siRNA targeting *TBK1* (*siTBK1*) or scrambled RNA (*siSCRAMBLE*) and subsequently infected with eGFP-CVB at MOI 0.01. (A) Fluorescence microscopy of infected HeLas at 24 h postinfection (p.i.). Phase contrast images show similar cell density at 24 h p.i. Scale bars represent 100 μm. (B) Extracellular viral titers of infected cells at 24 h as measured by plaque assay. **, *p* < 0.01, Student *t* test; n = 3. (C) Western blots of infected cells at 0 h, 6 h, and 24 h p.i. including densitometry. A representative Western blot is shown. **, *p* < 0.01, Student *t* test; n = 8. Data are representative of 4 experiments. WL = whole lysate.

We further confirmed these results using a HeLa cell model by silencing *TBK1* using siRNA. Since HeLa cells are more susceptible to CVB infection than MEFs, we infected cells at MOI 0.01 and harvested cells at 0 h, 6 h, and 24 h p.i. We observed substantial increases in viral eGFP positivity (36% increase; *, *p* < 0.05) (Fig 2A) and viral release (Fig 2B) in *TBK1*-silenced cells (*siTBK1)* compared to cells transfected with scrambled RNA (*siSCRAMBLE*), indicating greater viral production during *TBK1* silencing. Additionally, we observed higher intracellular VP1 levels in *siTBK1* HeLa cells (Fig 2C) although the difference was modest compared to the findings with MEFs. The difference in intracellular viral titers and extracellular viral titers in *siTBK1* cells could only be observed with a low infection concentration requiring successive rounds of infection in a multi-step measurement of viral replication (S2A Fig). Multi-step growth curves amplify subtle phenotypic effects over multiple replication cycles using low MOI [27]. When infecting with a high MOI of virus in a single-step measurement of viral replication over a single replication cycle, differences in infection could no longer be observed early in infection (S2B Fig). This may suggest that TBK1 does not affect permissivity to CVB infection, but instead primarily affects viral egress in HeLa cells (S2B Fig). Additionally, infection at MOI 0.1 for 6 hours showed no differences in eGFP expression (> 1% decrease; not significant) nor viral capsid VP1 by Western Blot (S2C and S2D Fig). These data highlight that TBK1 could potentially play a role in limiting viral spread after initial infection.

### Knockdown of TBK1 impairs autophagic flux

**Fig 3:**
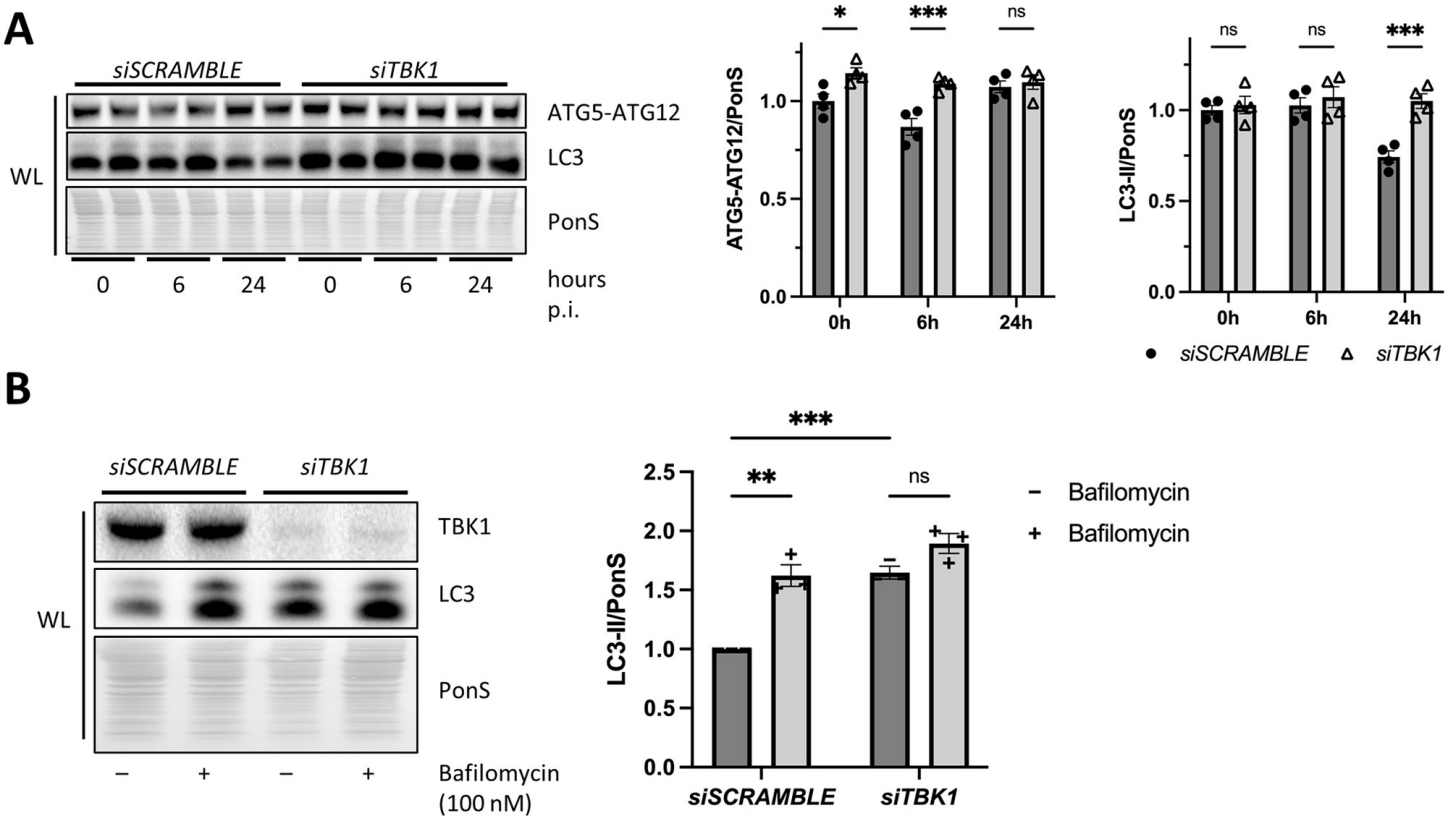
Silencing *TBK1* impairs autophagic flux. HeLa cells were treated with siRNA targeting *TBK1* (*siTBK1*) or scrambled RNA (*siSCRAMBLE*). (A) Western blot of infected cells at 0 h, 6 h, and 24 h p.i. at MOI 0.01. Densitometric quantification of Western blots. *, *p* < 0.05, ***, *p* < 0.001, two-way ANOVA; n = 4. (B) Western blot of cells treated with vehicle or bafilomycin (100 nM) for 2 h. Densitometric quantification of Western blots. **, *p* < 0.01, ***, *p* < 0.001, two-way ANOVA; n = 3. Data are representative of 3 experiments. WL = whole lysate.

Due to the reported role of TBK1 in autophagosome formation and autolysosomal fusion, we next sought to determine the consequences of *TBK1* silencing on autophagy. Following *TBK1* silencing, we infected cells at MOI 0.01 over a time course of 0, 6, and 24 h to observe the dynamics of viral infection over time. With *TBK1* silencing, we observed an increase in autophagosome formation as indicated by elevated ATG5-ATG12 conjugation at 0 and 6 h p.i. [21,22]. Interestingly, LC3-II was maintained at a high level in *siTBK1* cells compared to *siSCRAMBLE* controls indicating a potential block in autophagic flux (Fig 3A). To confirm this, we performed an autophagic flux assay to distinguish if the accumulation in LC3-II was due to reduced autophagosome-lysosome fusion. We treated *siSCRAMBLE or siTBK1* cells with bafilomycin and measured the lipidation of LC3. Bafilomycin is a lysosomal proton pump inhibitor that is known to block autophagic flux [28]. As expected, in *siSCRAMBLE* cells, bafilomycin induced a marked elevation of LC3-II (60%) consistent with autophagosome accumulation (Fig 3B). However, in *siTBK1* cells, the starting level of LC3-II was already elevated, and the addition of bafilomycin caused only a modest increase (12%), suggesting impaired flux with silencing of *TBK1*.

### Silencing TBK1 increases EV release

Our findings thus far have shown that loss of TBK1 increases CVB infection and disrupts autophagic flux. It is unclear if these two observations occur independently of each other or work in concert to increase viral infection and consequently, viral propagation. Our group previously described the release of viral EVs displaying both autophagosomal and mitophagosomal markers [10]. These EVs appeared to derive from fragmented autophagosome-enveloped mitochondria and contained infectious virions and pro-viral microRNAs [14]. Therefore, we investigated whether EV release may be impacted during *TBK1* knockdown.

We first isolated EVs from cell culture supernatant clarified of dead cells and debris using ExoQuick-TC and measured viral titers in free virus versus EV virus. We confirmed that EVs isolated with Exoquick-TC expressed common EV markers including ALIX, flotillin-1, and CD63 (S3A Fig). Further, to confirm that the virus is inside EVs, we performed a co-IP on EV isolates to pulldown flotillin-1 (S3B Fig). In addition to CD63, VP1 also co-precipitated with flotillin-1 in EVs from CVB-infected cells, showing that indeed the virus was associated with EVs. We next immunoprecipitated reticulon-3, an intracellular membrane protein located on the endoplasmic reticulum, with VP1 (S3C Fig). VP1 only co-precipitated with reticulon-3 in the infected cell lysate, indicating that the viral protein actively secreted in EVs was not associated with lysed intracellular remnants.

**Fig 4:**
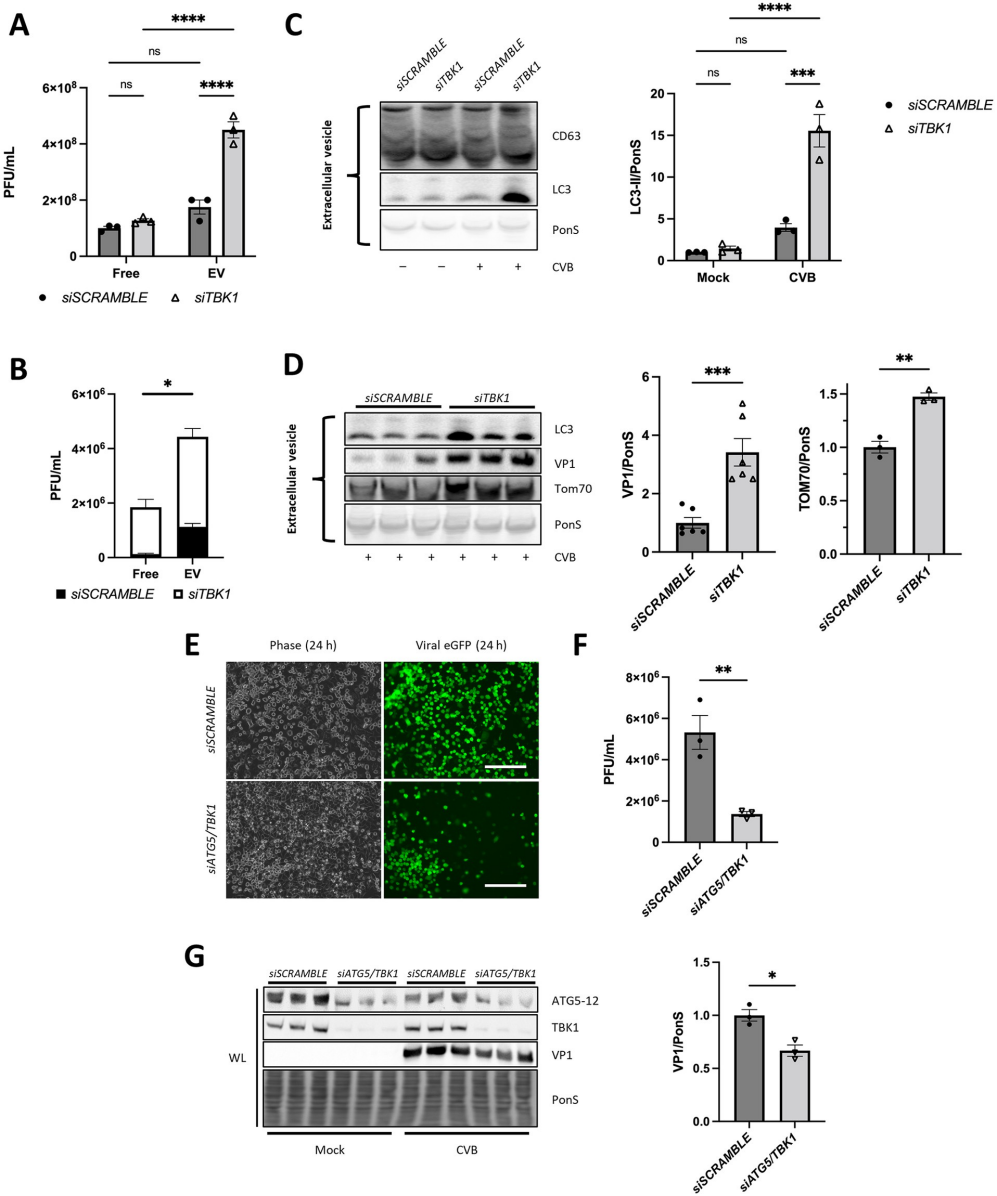
Loss of *TBK1* increases the release of infectious extracellular vesicles. (A) HeLa cells were infected with eGFP-CVB at MOI 0.1. Plaque assays on free virus (free, isolated from Exoquick-TC supernatant) and EV virus (EV) isolated from cells 24 h p.i. ****, *p* < 0.0001, two-way ANOVA; n = 3. (B) Ratio of free virus to EV virus plaque forming units shown in (A). *, *p* < 0.05, Student *t* test; n = 3. (C) Western blots of EV lysates isolated from mock-infected and CVB-infected cells at 24 h p.i. including densitometry. ***, *p* < 0.001, two-way ANOVA; n = 3. (D) Western blots of EV lysates from CVB-infected cells at 24 h p.i. including densitometry. **, *p* < 0.01, ***, *p* < 0.001 two-way ANOVA; n = 3. (E) ATG5 and TBK1 were silenced in HeLa cells then infected at MOI 0.01 for 24 h. Fluorescence microscopy images of cells at 24 h p.i. Phase contrast images show similar cell density. Scale bars represent 100 μm. (G) Extracellular viral titers of cell supernatants as measured by plaque assay. **, *p* < 0.01, Student *t* test; n-3. (G) Western blots on WL from mock verus infected cells at 24 h p.i. *, *p* < 0.05, Student *t* test; n = 3. Data are representative of 3 experiments. WL = whole lysate.

Next, we isolated EVs from *TBK1*-knockdown cells. Plaque assays revealed that EV virus was increased by ~200% when *TBK1* was silenced compared to controls (Fig 4A). Strikingly, the ratio of virus in EVs vs free virus remaining from the supernatant was significantly increased during *TBK1*-silencing which supports our hypothesis that TBK1 limits viral-EV spread (Fig 4B). We further interrogated the protein content of these EVs by western blot. By comparing equivalent protein concentrations for each EV sample, we observed that EVs shed from mock or infected cells contained similar amounts of CD63 (Fig 4C). However, we found that EVs shed from *siTBK1* cells were much more enriched with LC3-II compared to EVs from *siSCRAMBLE* cells during infection (Fig 4C). EVs isolated from *siTBK1* cells also contained significantly more VP1 which is consistent with the increased viral titers (Fig 4D). As mentioned, we had reported that CVB-induced EVs could also derive from virus-containing mitophagosomes, and indeed we observe that EVs from infected *siTBK1* cells also contained increased amounts of mitochondrial outer membrane protein TOM70. These results suggest that loss of TBK1 in the setting of CVB infection leads to increased release of virus-laden EVs that may be originating from mitophagosomes.

To conclude that CVB is degraded via autophagy and thereby prevents CVB release through EVs, we performed a double silencing of *ATG5* and *TBK1*. ATG5 is an indispensable factor for autophagic vesicle formation, whereby knockdown of *ATG5* inhibits autophagy [22,29]. It has been shown that CVB utilizes autophagy machinery to replicate on the autophagosome membrane, therefore it is expected that knockdown of *ATG5* would inhibit viral replication [10,30–33]. We silenced *ATG5* and *TBK1* (*siATG5/TBK1*) in HeLa cells then infected them at MOI 0.01 for 24 h. By fluorescence microscopy, we observed a reduction in eGFP-positive cells in *siATG5/TBK1* cells (60% decrease; **, *p < 0*.*01*) (Fig 4E) and a significant reduction in extracellular viral titers (Fig 4F). Finally, Western blots confirmed a significant decrease in cellular VP1 (Fig 4G). In all, these data depict the complex relationship between CVB replication, egress, and elimination.

### CVB infection is unaltered during IFNAR knockdown

**Fig 5:**
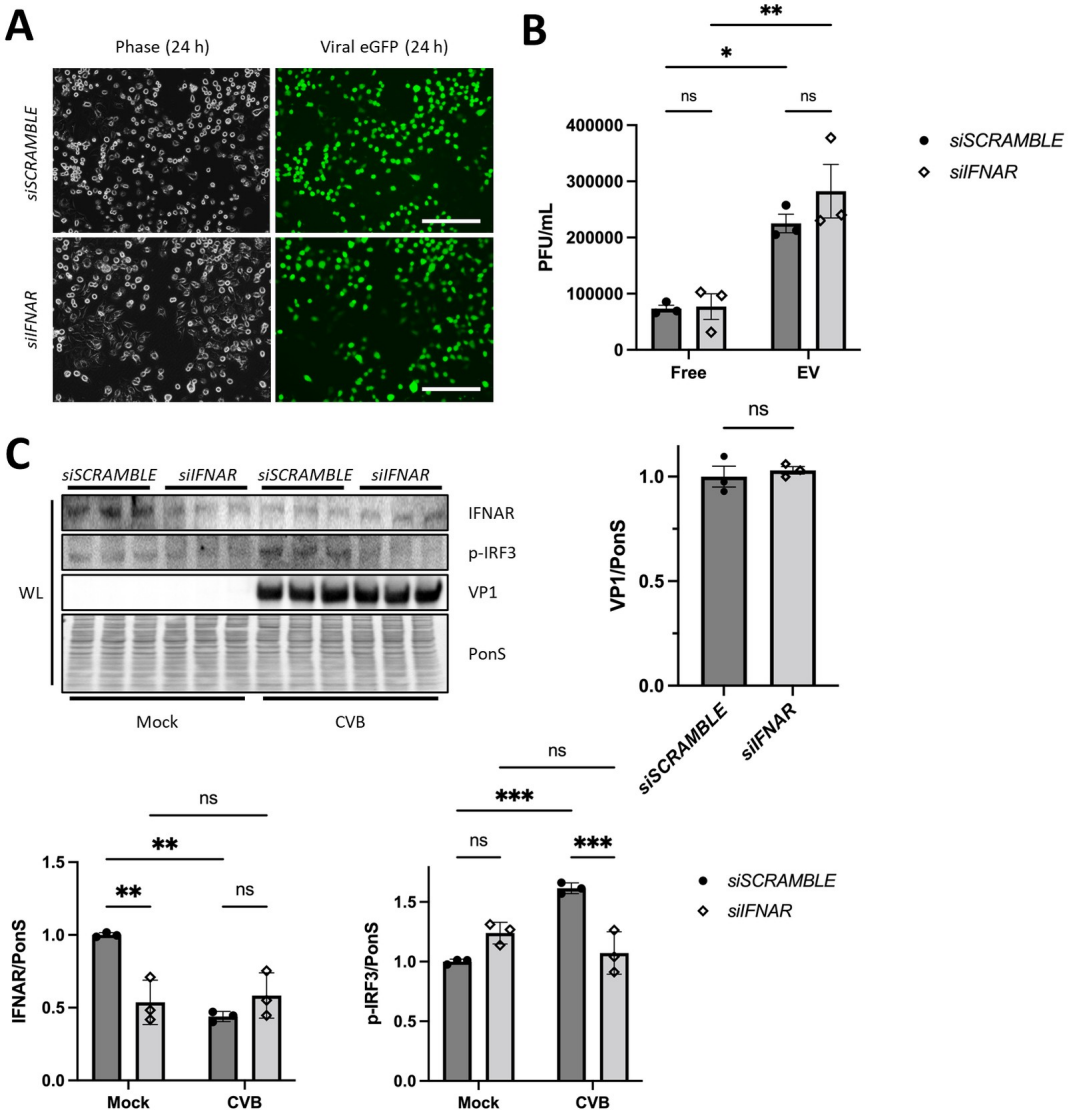
Silencing *IFNAR* does not alter CVB infection. HeLa cells were treated with siRNA targeting *IFNAR* (*siIFNAR*) or scrambled RNA (*siSCRAMBLE*) and subsequently infected with eGFP-CVB at MOI 0.01. (A) Fluorescence microscopy of infected HeLas at 24 h postinfection (p.i.). Phase contrast images show similar cell density at 24 h p.i. Scale bars represent 100 μm. (B) Extracellular viral titers of infected cells at 24 h as measured by plaque assay. *, *p* < 0.05, **, *p* < 0.01; two-way ANOVA; n = 3. (C) Western blots of infected cells at 24 h p.i. including densitometry. **, *p* < 0.01, ***, *p* < 0.001, Student *t* test or two-way ANOVA; n = 3. Data are representative of 2 experiments. WL = whole lysate.

To understand if the observed increase in viral infection during *TBK1*-deficiency is a consequence of type I interferon response inhibition, we examined viral infection in interferon-alpha/beta receptor (*IFNAR*)-silenced cells. Because TBK1 mediates the phosphorylation and nuclear transcription of IRF3 during infection [34], we also examined activation of IRF3. We silenced *IFNAR* in HeLa cells and infected them at MOI 0.01 for 24 h. No differences were observed in regard to viral eGFP expression (7% decrease; not significant) (Fig 5A) and levels of both extracellular free virus and EV virus was similar between *siSCRAMBLE* controls and *siIFNAR* cells (Fig 5B). Although Western blot revealed knockdown of IFNAR and reduction in IRF3 phosphorylation at 24 h p.i., no remarkable differences in VP1 were seen (Fig 5C). Though not significant, there was a small trend towards increased infection to in *siIFNAR* cells. Thus, this data supports our hypothesis that TBK1 preferentially exerts its antiviral role through autophagy. These data correspond with previous reports that CVB does not produce a significant interferon response [7].

### Activating TBK1 limits CVB infection and propagation via EVs

**Fig 6:**
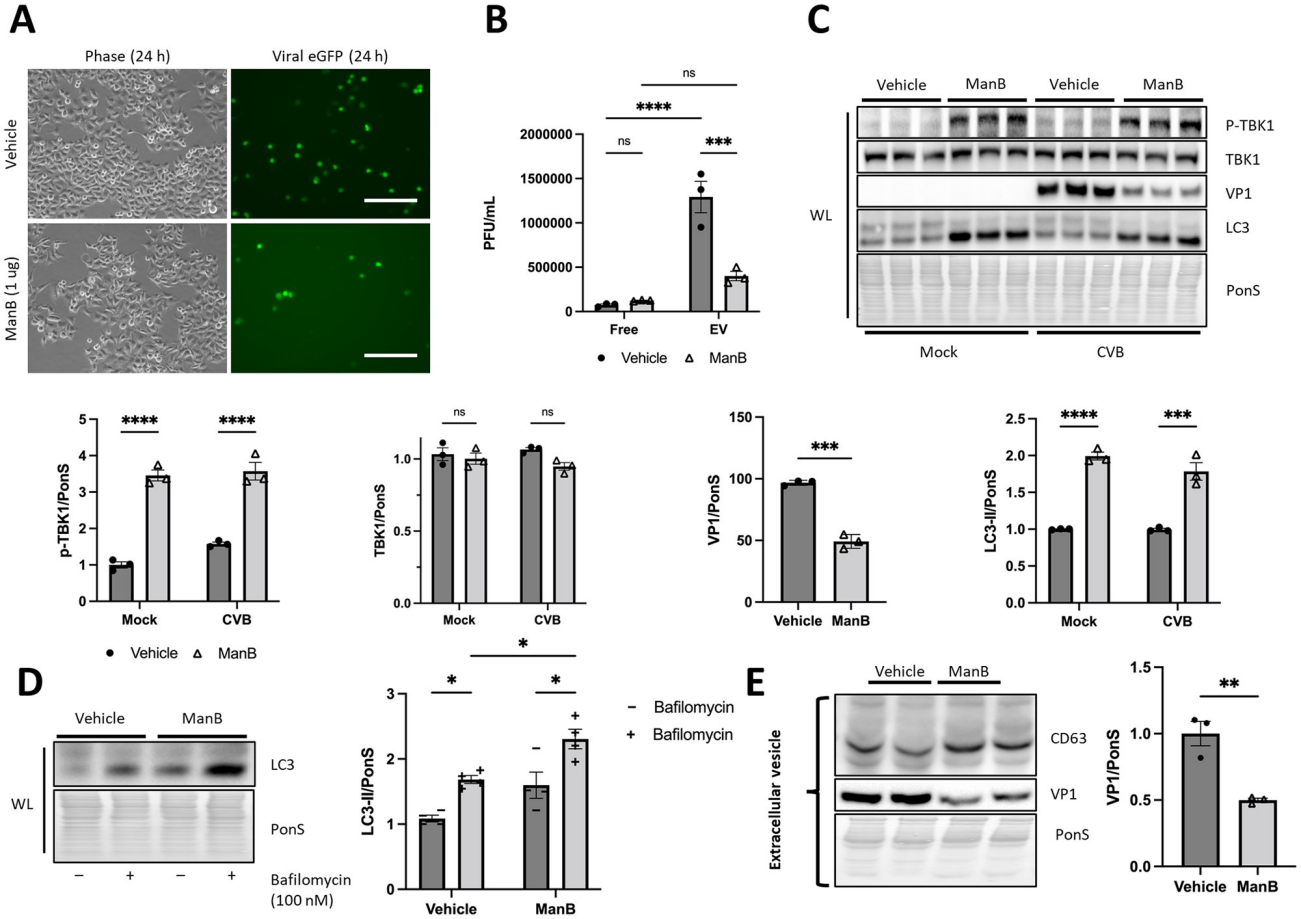
Activating *TBK1* with Manassantin B suppresses CVB infection and secretion into EVs. HeLa cells were treated with 1 μg ManB 24 h prior to infection with eGFP-CVB for an additional 24 h. (A) Fluorescence microscopy images of cells infected at MOI 0.01 for 24 h. Phase contrast images show similar cell density at 24 h p.i. Scale bars represent 100 μm. (B) Extracellular viral titers of cells infected at MOI 0.1 for 24 h as measured by plaque assay. ***, *p* < 0.001, ****, *p* < 0.0001, two-way ANOVA; n = 3. (C) Western blots of mock-infected and CVB-infected cells at MOI 0.01 for 24 h. Densitometric quantification of Western blots. ***, *p* < 0.001, ****, *p* < 0.0001, two-way ANOVA or Student *t* test; n = 3. (D) Western blot of cells treated with bafilomycin (100 nM) for 2 h following treatment with ManB (1 ug) or vehicle for 24 h. Densitometric quantification of LC3-II. *, *p* < 0.05, two-way ANOVA; n = 4. (E) Western blot of EV lysates from CVB-infected cells including densitometric quantification of VP1. **, *p* < 0.01, Student *t* test; n = 3. Data are representative of 3 experiments. WL = whole lysate.

We have so far demonstrated that loss of TBK1 impairs autophagic flux and promotes the release of infectious EVs. This suggests that TBK1 plays a novel role in viral elimination. To further interrogate this, we treated HeLa cells with the specific TBK1 agonist Manassantin B (ManB) to test effects on CVB infection and viral EV shedding. ManB is a neolignan isolated from *Saururus chinensis* that exhibits antiviral and anti-inflammatory effects via activation of the STING-TBK1 pathway [25]. After infecting with eGFP-CVB for 24 h, we saw a marked reduction in eGFP cells following ManB treatment (14% decrease; *, *p* < 0.05) (Fig 6A) and plaque assays revealed a substantial reduction in EV-associated viral titers but interestingly, free viral titers were not significantly different (Fig 6B). This may indicate that TBK1 upregulation inhibits CVB egress primarily by blocking viral EV release. We also observed a reduction in cellular VP1; however, we also observed a significant increase in LC3-II (Fig 6C). To understand if this increase was due to increased autophagy or a block in autophagic flux, we incubated vehicle or ManB-treated cells with bafilomycin. We found that bafilomycin increased accumulation of lipidated LC3 to a greater extent in ManB-treated cells than vehicle-treated cells, suggesting activating TBK1 with ManB increases autophagic flux (Fig 6D). Importantly, western blots of isolated EVs from ManB-treated cells showed a dramatic reduction in VP1 compared to vehicle-treated cells (Fig 6E). These findings highlight a key role for TBK1 in degradation of CVB via autophagy, thereby preventing CVB release via EVs.

### GABARAPL1/2 facilitate viral degradation

To further elucidate the formation of viral EVs, we examined additional autophagy-related targets of TBK1 including GABARAP family members [24]. The ATG8 proteins (including LC3, GABARAP, and their subfamily members) each have a unique role in orchestrating membrane-trafficking events. LC3 is widely used as an indication of autophagy; the lipidated form (LC3-II) is bound to the autophagosome by conjugation to membrane phosphatidylethanolamine (PE) and functions in autophagosome biogenesis and substrate selection. GABARAP and the closely related paralogs GABARAPL1 and GABARAPL2, are cytosolic proteins that participate in autophagosome initiation and interact with cargo adaptor molecules (such as p62/SQSTM1) [35]; they also participate in vesicle transport via microtubules [36]. Although GABARAP family members are less-studied than LC3, studies have demonstrated their active role in facilitating insulin secretion, GABA(A) receptor trafficking, and angiotensin II type 1 receptor trafficking to the plasma membrane [37–39]. Therefore, we chose to examine the effect of GABARAP family members on EV shedding because of their known role in autophagosome trafficking and potential role in EV release.

**Fig 7:**
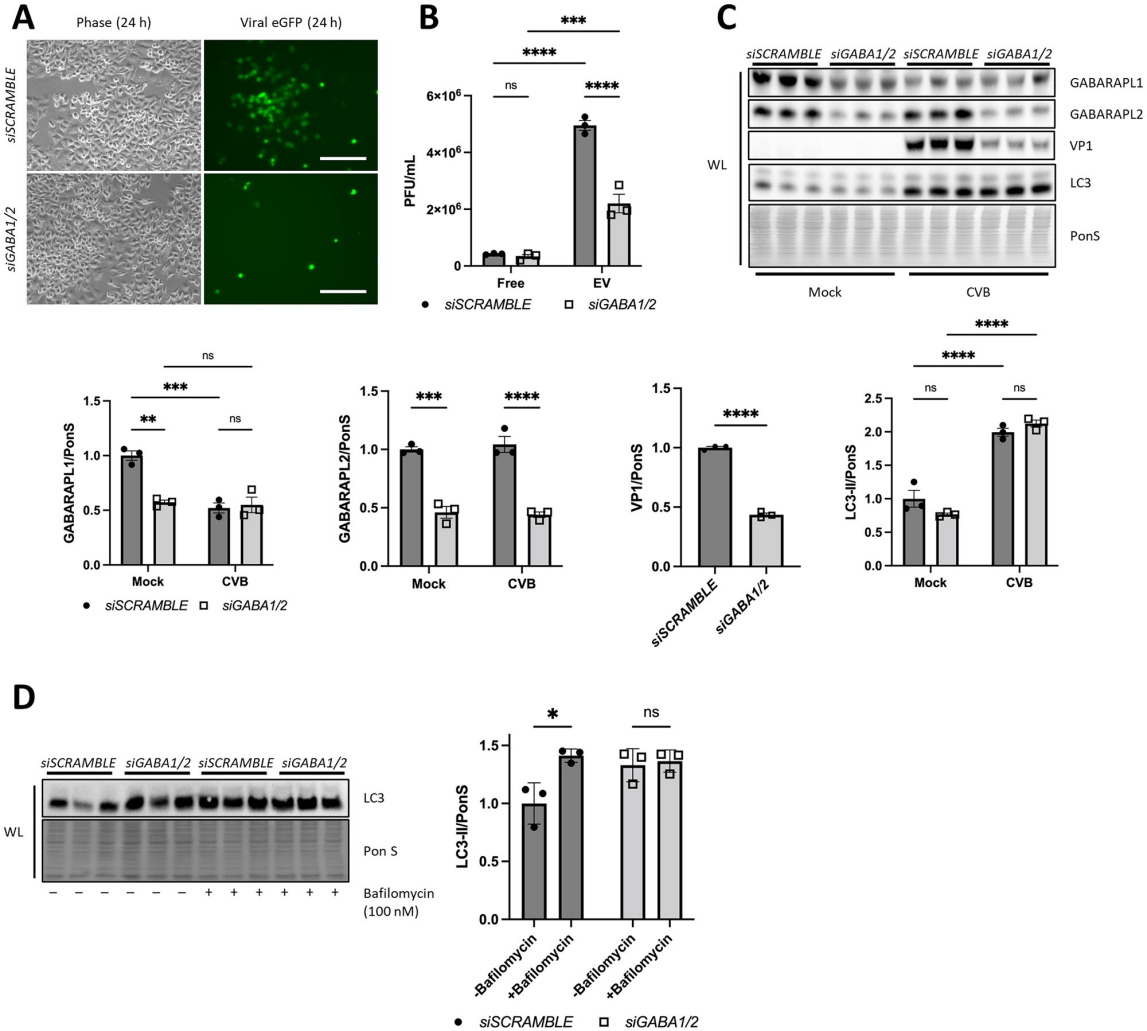
Silencing *GABARAPL1* and *GABARAPL2* reduces CVB infection and secretion into EVs. (A) HeLa cells were treated with siRNA targeting *GABARAPL1* and *GABARAPL2* (*siGABA1/2*) and subsequently infected with eGFP-CVB for 24 h. Fluorescence microscopy of HeLa cells infected at 24 h p.i. at MOI 0.01. Phase contrast images show similar cell density at 24 h p.i. Scale bars represent 100 μm. (B) Extracellular viral titers of free virus (free) or EV virus (EV) at 24 h p.i. at MOI 0.1 as measured by plaque assay. ***, *p* < 0.001, ****, *p* < 0.0001, two-way ANOVA; n = 3. (C) Western blots of infected cells at 24 h p.i. at MOI 0.01. Densitometric quantification of Western blots. **, *p* < 0.01, ***, *p* < 0.001, ****, *p* < 0.0001, two-way ANOVA; n = 3. (D) *siSCRAMBLE* or *siGABA1/2* cells were treated with vehicle or bafilomycin (100 nM) for 2 h. Cell lysates were analyzed by Western blot and densitometry quantification was performed. *, *p* < 0.05, two-way ANOVA; n = 3. Data are representative of 3 experiments. WL = whole lysate.

First, we identified whether EVs isolated using the ExoQuick-TC system contained GABARAP family members. We analyzed EV lysates from *siSCRAMBLE* vs. *siTBK1* cells. Strikingly, GABARAPL1 and GABARAPL2 were expressed at higher levels in *siTBK1* EVs compared to *siSCRAMBLE* EVs in CVB-infected groups (S4 Fig). To definitively identify the role of GABARAP family members during viral infection, we performed individual silencing of *GABARAPL1* or *GABARAPL2* in HeLa cells (S5 Fig). We did not observe a difference in infection in either of the groups at 24 h p.i. This led us to perform a double-knockdown experiment using both siRNAs. Remarkably, infection was strongly reduced at 24 h p.i. as observed by eGFP expression (37% decrease; *, *p* < 0.05), and infectious EV release was also decreased (Fig 7A–7D). Interestingly, CVB reduced cellular levels of GABARAPL1 24 h p.i. (Fig 7C). Of note, silencing both *GABARAPL1 and GABARAPL2* (*GABA1*/2) did not alter LC3 compared to *siSCRAMBLE* cells (Fig 7C) which strongly reveals the importance of GABARAP family members in maintaining autophagic flux. To confirm whether GABARAP family members are important to maintain autophagic flux, we treated *GABA1/2*-silenced cells with bafilomycin. Lipidation of LC3-II was significantly increased in *siSCRAMBLE* cells (~41%) compared to *siGABA1/2* (~3%), leading us to conclude that GABARAP family members are critical in maintaining flux (Fig 7D). The observed increase in LC3-II despite limited viral replication in *siGABA1*/2 cells appears to be independent of productive infection as demonstrated by a slight increase in LC3-II during infection of HeLa cells with UV-inactivated or heat-inactivated CVB in both whole cell and EV lysates (S6 Fig).

We have shown that knocking down TBK1 enhances CVB release through EVs, and we hypothesized this is due to disruption in GABA1/2-mediated autophagic flux. Based on recent literature that shows that TBK1 directly activates GABARAPL2 through phosphorylation to control autophagosome shedding and that secretion of EVs is dependent on GABARAPL1 [24,40], we hypothesize that TBK1 activates GABARAPL1 and GABARAPL2 to coordinate viral degradation in the host cells by chauffeuring virus-laden autophagosomes to the lysosome to be degraded. Because GABARAPL1 and GABARAPL2 are also required for autophagosome closure, we further hypothesize that silencing GABA1/2 restricts CVB infection early on by limiting the completion of autophagosome structures used by CVB for replication [41–43]. For the first time, these data illuminate a novel and fundamental role for autophagy modifiers GABARAPL1 and GABARAPL2 in viral elimination.

### Amlexanox treatment worsens viral pancreatitis and increases viral EVs in serum

**Fig 8:**
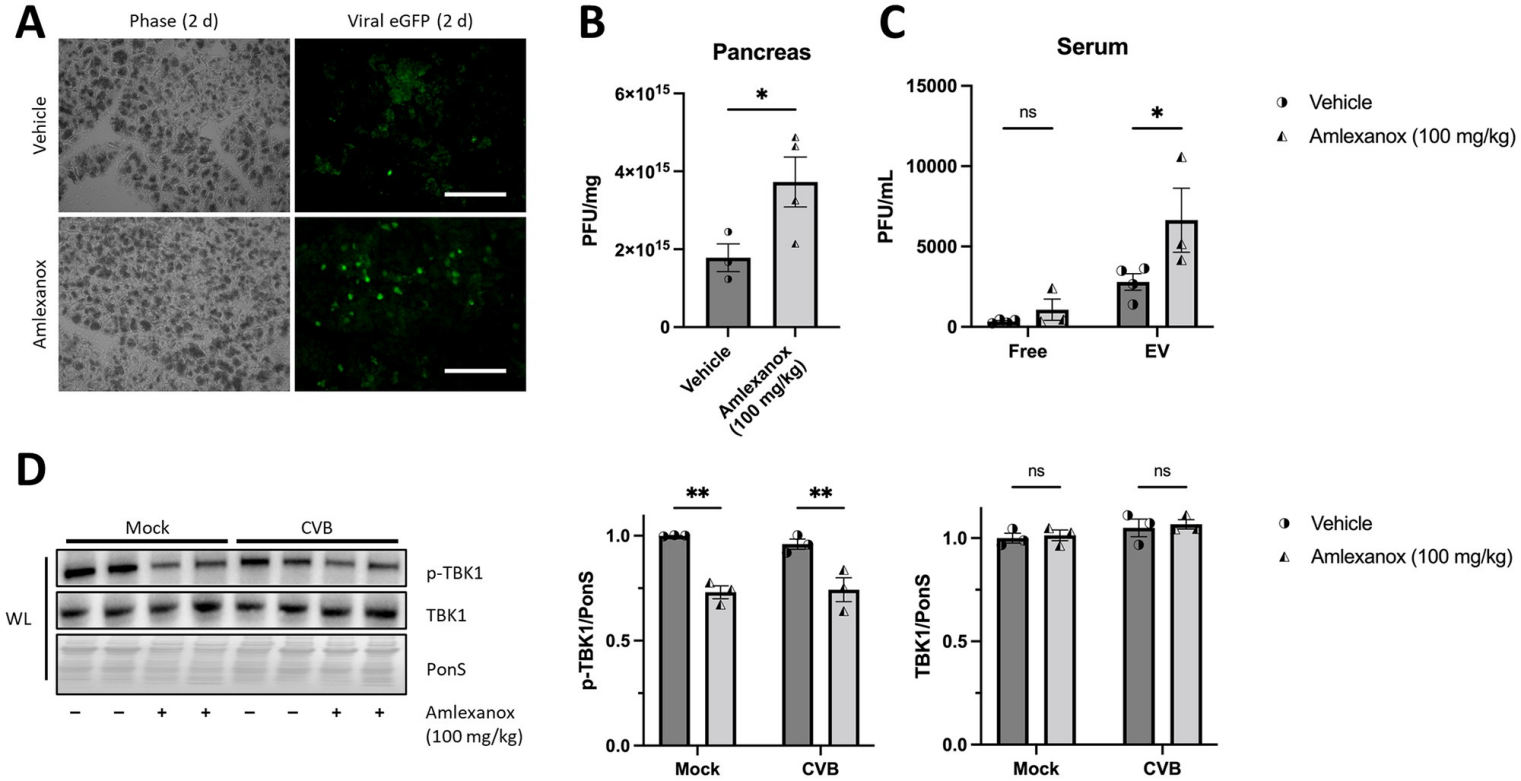
*In vivo* Amlexanox treatment increases pancreatic CVB titers and serum EV titers in CVB-infected mice. Ten-week-old C75BL/6 male mice were treated with 100 mg/kg Amlexanox or equal volume vehicle for 5 days pre-infection and each day following infection via oral gavage. On the fifth day of Amlexanox treatment, mice were infected I.P. with 10^7^ plaque forming units (PFU) eGFP-CVB. (A) Fluorescence microscopy of cryo-frozen pancreata at 3d p.i. (B) Pancreatic viral titers as measured by plaque assays on pancreatic homogenates. *, *p* < 0.05, Student *t* test; n = 3–4. (C) Isolated free or EV viral titers as measured by plaque assays from serum. *, *p* < 0.05, two-way ANOVA; n = 3–4. Data are representative of 3 experiments. (D) Western blot of mouse pancreata isolated at 2 days p.i. **, *p* < 0.01, two-way ANOVA; n = 3–4. WL = whole lysate.

**Fig 9:**
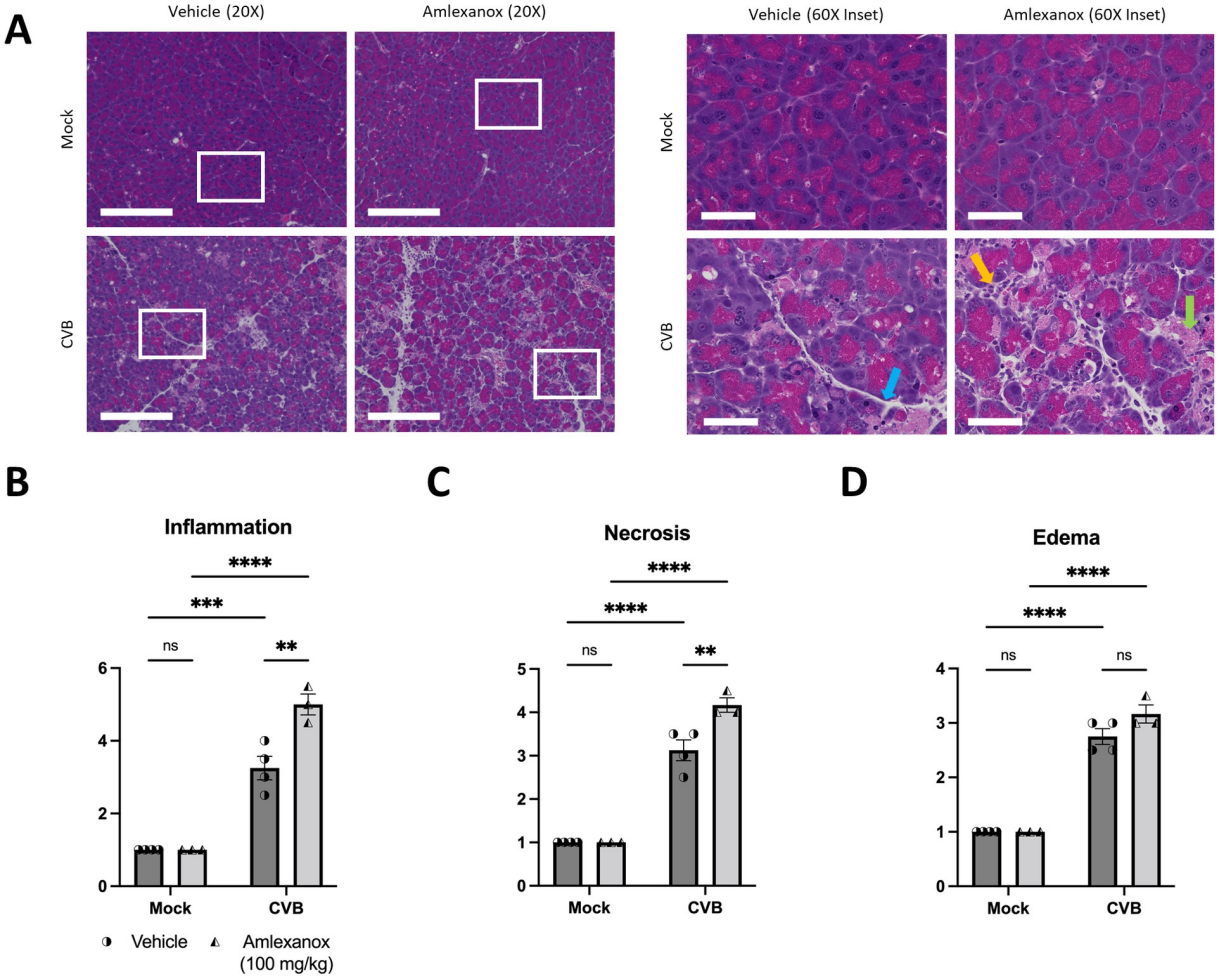
*In vivo* Amlexanox treatment increases pancreatic tissue destruction in CVB-infected mice. Ten-week-old C75BL/6 male mice were treated with 100 mg/kg Amlexanox or equal volume vehicle for 5 days pre-infection and each day following infection via oral gavage. On the fifth day of Amlexanox treatment, mice were infected I.P. with 10^7^ plaque forming units (PFU) eGFP-CVB. (A) Hematoxylin and eosin staining on representative pancreas sections. Scale bars represent 50 μm. (B) Quantification of inflammation (yellow arrow) in mock-infected versus CVB-infected pancreata with or without Amelxanox treatment. (C) Quantification of necrosis (green arrow) in mock-infected versus CVB-infected pancreata with or without Amelxanox treatment. (D) Quantification of edema (blue arrow) in mock-infected versus CVB-infected pancreata with or without Amelxanox treatment. All tissue sections were scored blindly. **, *p* < 0.01, ***, *p* < 0.001, ****, *p* < 0.0001, two-way ANOVA; n = 3–4. Data are representative of 3 experiments.

Having demonstrated a new role for TBK1 in attenuating the release of viral EVs in cells, we sought to confirm these findings *in vivo*. We used a mouse model of viral pancreatitis for our studies to explore the effect of TBK1 inhibition on CVB infection because the pancreas is an early target for viral infection during acute infection. To do this, we pre-treated mice with 100 mg/kg Amlexanox, a specific inhibitor of TBK1 and its homolog IKKε, for 5 days prior to eGFP-CVB infection and every day following infection. We infected mice with 10^7^ plaque forming units (PFU) CVB via intraperitoneal injection and isolated serum and pancreata two days p.i. Amlexanox treatment showed increased viral eGFP abundance in cryosections of pancreatic tissue (Fig 8A). Additionally, plaque assays on pancreatic homogenates revealed that viral titers were significantly increased in the pancreata of Amlexanox-treated animals (Fig 8B). We next sought to determine if Amlexanox could increase circulating levels of infectious viral EVs. Using ExoQuick-TC, we isolated EVs from serum isolated from animals 2 days p.i. and measured viral titers in the isolated EVs versus EV-depleted sera (Fig 8C). We observed that *in vivo* TBK1 inhibition by Amlexanox treatment markedly increased infectious EV levels, but not free virus levels in the sera. Western blots of pancreatic lysates revealed reduced TBK1 phosphorylation, suggesting impaired TBK1 activity (Fig 8D). Further, hematoxylin and eosin staining of pancreatic sections revealed more necrosis, edema, and inflammatory cell infiltration in Amlexanox-treated mice compared to vehicle-treated mice (Fig 9A). Severity of pancreatic damage was blindly scored among treatment groups (Fig 9B–9D). Taken together, these data strongly establish a critical role for TBK1 in attenuation of viral spread.

## Discussion

Picornaviruses such as CVB had traditionally been thought to escape the host cell exclusively by inducing cytolysis and triggering the release of non-enveloped “naked” virions [44]. It was not until recent years when we and others had determined that several picornaviruses hijack intracellular membranes which eventually become expelled from the host cell as infectious virus-laden extracellular vesicles [11,12,45,46]. Our subsequent work further interrogated this phenomenon and had revealed that CVB associates with mitochondria, triggers mitophagy, and escapes the infected cell as virus-containing mitophagosomes [10]. Though this mode of vesicle-based viral egress is particularly unusual for non-enveloped viruses, it has been suggested that it could present a multitude of advantages for the virus [47,48]. In addition to prolonging host cell survival by circumventing the need for cytolytic viral egress, vesicle-based egress has been suggested to allow for *en bloc* transmission of viruses which allows for multiple virions to induce a productive infection [13,49]. Indeed, we had previously observed that virus within EVs displays increased infectivity compared to “free” naked virus [10,11].

Though much research has further characterized vesicle-based viral egress involving several more non-enveloped viruses, mechanisms by which these viruses become engulfed in intracellular membranes (autophagosomes in this study) and subsequently evade lysosomal degradation is yet unclear. It had previously been shown that CVB reduces levels of autophagosome-lysosome fusion proteins such as syntaxin-17 and SNAP29 in order to impair autophagic flux [50,51]. Non-enteroviruses such as Dengue virus have similarly been shown to target autophagy to prevent flux by degradation of p62/SQSTM1 and promote viral transmission via autophagy-like vesicles [52,53]. This unconventional modality of non-lytic viral secretion provides insight on the mechanisms behind EV formation and release.

In this study we describe the role of TBK1 in vesicle-based CVB egress. More specifically, we show that this member of the Iκκ family of kinases may inhibit release of viral extracellular vesicles by promoting autophagic flux. TBK1 has previously been shown to play roles in interferon signaling [17,34], autophagosome maturation [54–57], and autophagic flux [24,58]. We observe that *TBK1* knockout in primary MEFs or *TBK1* silencing in HeLa cells significantly increases viral protein production and viral release (Figs 1 and 2). We verify that genetic knockout of *TBK1* limits cellular viral titers as determined by plaque assays on cell lysates (S1 and S2 Figs).

As a significant and active kinase in autophagy, TBK1 is demonstrated to be essential to maintain autophagic flux (Fig 3). Interestingly, though free virion release is not significantly altered in Hela-infected cells following *TBK1* silencing, the release of virus within EVs is dramatically increased (Fig 4). These vesicles from *TBK1*-silenced infected cells are greatly enriched with LC3 and the mitochondrial outer membrane protein TOM70, which is consistent with the notion that TBK1 plays roles in inhibiting virus-laden auto/mitophagosome formation and release as EVs. We verify that viral replication and extracellular release is dependent on the autophagy pathway through *ATG5/TBK1* silencing (Fig 4). When we inhibited the interferon-alpha/beta receptor (IFNAR), we found that silencing *IFNAR* does not significantly alter infection (Fig 5), confirming our hypothesis that TBK1 mainly exerts its anti-CVB role through the autophagy pathway. In agreement, activation of TBK1 with Manassantin B limits CVB infection by suppressing viral vesicle release (Fig 6).

Our discovery that these EVs also contained GABARAPL1 and GABARAPL2 demonstrate another novel therapeutic strategy to block CVB infection (S4 Fig). We found that simultaneously silencing the ATG8 family members GABARAPL1 and GABARAPL2 dramatically suppressed CVB infection, which was marked by statistically significant reductions in both intracellular and extracellular virus (Fig 7). When we examined lysates from infected cells, we found that CVB infection indeed reduced levels of GABARAPL1, raising the question if CVB targets GABARAP family members to escape host immunity and promote subsequent rounds of infection. Early research has identified proteolytic targets of CVB including mitochondrial antiviral signaling (MAVS) and TRIF to attenuate host antiviral response [7], however there are additional gaps in our knowledge of viral targets that remain to be studied. GABARAP family members have been implicated in the pathogenesis of HIV-1 [59]. Researchers discovered that HIV-1 Nef, an accessory protein encoded by HIV, directly binds to GABARAPs independent of LC3 for plasma membrane localization [60,61]. Compromised anterograde trafficking in the absence of GABARAPs was recently reported to alter the morphology of the Golgi due to inhibited vesicular trafficking of lipids [62]. Thus, our observation that GABARAP family members also facilitate viral infection describe an opportunistic relationship between CVB and autophagy for survival.

Finally, we show that treating mice with the TBK1 inhibitor Amlexanox prior to CVB infection significantly elevates pancreatic viral titers, as well as serum levels of virus within EVs while not altering circulating levels of free virus (Fig 8). Amlexanox also significantly exacerbates CVB-mediated pancreatic inflammation, necrosis and edema in those mice as well (Fig 9). In addition to bolstered viral packing of EVs in serum, we postulate that inhibition with Amlexanox further reduces interferon-based responses to CVB infection potentially worsening tissue destruction. Though Amlexanox is clinically used as an anti-inflammatory for the treatment of ulcers [63,64] and asthma [65], we present compelling data showing its immunosuppressive potential during viral infection. These data highlight the immediate importance of research into modes of viral dissemination and potential biomarker targets to potentially detect viral infection.

**Fig 10:**
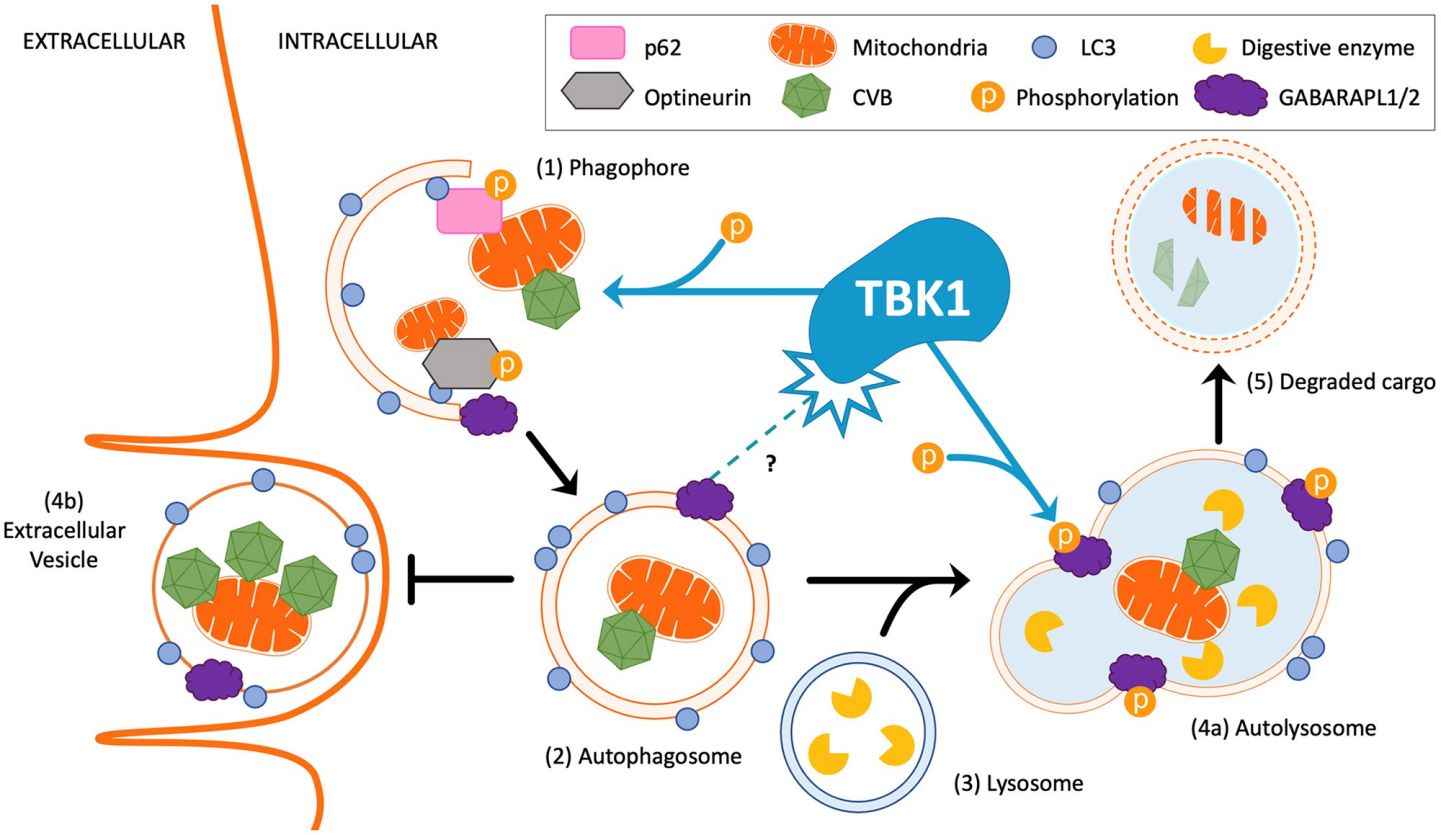
Hypothetical model of TBK1 function and CVB infection. CVB has previously been shown to rely on autophagy to promote viral replication. Here, we describe the anti-viral role of the phosphor-kinase TBK1 in autophagic elimination of CVB. (1) Phagophore initiation is by the recruitment and assembly of autophagic machinery. Among the key machinery components, ATG8 family members including LC3, GABARAPL1, and GABARAPL2 regulate phagophore elongation. ATG8 proteins coordinate cargo selection (such as damaged mitochondria and pathogens like CVB) with autophagy adaptors, p62 and Optineurin. TBK1 is known to directly phosphorylate p62 and Optineurin for efficient cargo selection. (2) Upon closure of the phagophore, the autophagosome is formed and fuses with the (3) lysosome to form the autolysosome. Here, TBK1 phosphorylates LC3 and GABARAPL2 to inhibit premature shedding of the autophagosome and promote flux. (4a) Upon fusion, the inner membrane space of the autolysosome undergoes acidification and digestive enzymes are activated to (5) degrade its contents. (4b) However, in the absence of TBK1, the autophagosome subverts fusion with the lysosomes and is released from the intracellular space to the extracellular space as an extracellular vesicle (EV) containing infectious CVB and mitochondrial fragments.

Though TBK1 has been implicated in suppression of CVB infection via interferon signaling, our findings demonstrate an additional critical antiviral role for TBK1 in the suppression of viral replication and vesicle-based viral egress through autophagy [25,66]. We hypothesize that TBK1 facilitates autophagic degradation of virus-laden autophagosomes before they can be released from the cell as EVs (Fig 10). Blocking TBK1 activity may enable CVB to accumulate within autophagosomes and disrupt their fusion with the lysosome. The multi-faceted antiviral nature of TBK1 make it a very promising therapeutic target to limit viral replication and spread; thus, additional studies must be done to further characterize *in vivo* use of TBK1-activating agents to limit CVB infection and subsequent viral disease.

## Materials and methods

### Ethics statement

All mouse work adhered to the National Institutes of Health guidelines and was approved by Cedars-Sinai Medical Center’s Institutional Animal Care and Use Committee (IACUC008471, 1 February 2019–31 January 2022). Prior to sacrifice, animals were first anesthetized with isoflurane and underwent cervical dislocation.

### Production of recombinant coxsackievirus

The production of recombinant coxsackievirus B3 (pMKS1) expressing protein inserts has been previously described [67]. Briefly, an infectious CVB3 clone (pH 3) was engineered with a unique SfiI restriction site allowing for the insertion of foreign DNA fragments. Enhanced green fluorescent protein (eGFP) and timer protein were amplified from expression plasmids using sequence-specific primers with flanking SfiI sequences. PCR products were cloned into pMKS1 to generate eGFP-CVB and timer-CVB. Constructs were transfected into HeLa RW cells maintained in Dulbecco’s modified Eagle’s medium (DMEM; Gibco; 11995–073), and infectious virus was produced. Cells were then scraped, freeze-thawed three times, and centrifuged at 2,000 rpm to remove cellular debris. Concentrations of viral stocks were determined by plaque assay.

### Cell culture and treatments

Wild-type mouse embryonic fibroblasts, TBK1^+/-^ mouse embryonic fibroblasts, TBK1^-/-^ mouse embryonic fibroblasts, and HeLa cervical cancer cells were maintained in DMEM growth medium consisting of DMEM supplemented with 10% fetal bovine serum (FBS; Life Technologies; 16010–159) and antibiotic/antimycotic (Life Technologies; 15240–062).

Bafilomycin A1 (Sigma-Aldrich; 19–148) was dissolved in dimethyl sulfoxide (DMSO) at a concentration of 100 mM and cells were treated at a concentration of 100 nM for the times indicated.

ATG5 siRNA (human [Santa Cruz Biotechnology; sc-41445]), IFNAR siRNA (human [Santa Cruz Biotechnology; sc-35637]), GABARAPL1 siRNA (human [Santa Cruz Biotechnology; sc-105386]), GABARAPL2 siRNA (human [Santa Cruz Biotechnology; sc-41958]), and TBK1 siRNA (human [Santa Cruz Biotechnology; sc-39058]) were reconstituted by following the manufacturer-provided datasheet. HeLa cells were transfected using Effectene transfection reagent by following the manufacturer’s guidelines for reagent volumes. Forty-eight hours following transfection, the medium was refreshed and cells were subsequently infected.

### EV isolation

EVs were isolated with ExoQuick-TC (Systems Biosciences; EXOTCxxA-1) as described previously. Briefly, medium was removed from infected cells and centrifuged at 3,000 × g for 15 min to clarify the media by pelleting cells and debris. Supernatant was transferred to new tube and ExoQuick-TC was added at a 1:5 dilution. After incubation overnight at 4°C, the mixture was centrifuged at 1,500 × g for 30 min to pellet EVs. Free virus was isolated from the Exoquick-TC supernatant. The pellet was then washed in phosphate-buffered saline (PBS) and then resuspended in DMEM, or lysed in radioimmunoprecipitation assay (RIPA) buffer containing Tris (pH 8.0) (50 mM; Sigma-Aldrich; T1503), NaCl (150 mM; Sigma-Aldrich; S7653), EGTA (1 mM; Sigma-Aldrich; E4884), NP-40 (1%; Sigma-Aldrich; I3021), sodium deoxycholate (0.5%; Sigma-Aldrich; D6750), SDS (0.1%; Bio-Rad Laboratories Inc.; 161–0302), and protease inhibitors (Sigma-Aldrich; 05056489001) pH adjusted to 7.4.

### Animal model

Animal ethics: All mouse work adhered to the National Institutes of Health guidelines and was approved by Cedars-Sinai Medical Center’s Institutional Animal Care and Use Committee (IACUC008697, 11 October 2019–30 September 2022). Prior to sacrifice, animals were first anesthetized with isoflurane and underwent cervical dislocation.

Amlexanox (Abcam; ab142825) treatments were prepared by dissolving Amlexanox in PBS containing 5% dimethyl sulfoxide (DMSO). The 10-week-old male C57BL/6J mice were treated with 100 mg/kg Amlexanox or equivalent volume vehicle via oral gavage six days prior to infection. On the sixth day, mice were infected with 10^7^ plaque forming units of EGFP-CVB with DMEM via intraperitoneal (IP) injection. The following day, mice were treated again with 100 mg/kg Amlexanox or vehicle. Two days post-infection, mice were sacrificed and pancreata and blood were harvested. Tissue was either flash frozen for plaque assay or fixed in 4% formaldehyde for histology. EVs were isolated from serum using ExoQuick-TC following the manufacturer’s protocol. For plaque assay, frozen pancreatic tissue was weighed and then homogenized in DMEM using a TissueLyzer LT instrument (Qiagen, Hilden, Germany). Homogenates were then clarified by centrifuging at 1000 × g for 10 min at 4 °C and the remaining supernatant was used.

### Histology

Fixed pancreatic tissue was embedded in paraffin and sectioned into 4 μm-thick sections. Tissue sections were then stained with hematoxylin and eosin. Sections were deparaffinized with xylene and rehydrated in decreasing concentrations of ethanol. Sections were stained with Gill 2 Hematoxylin (Richard-Allan Scientific, San Diego, CA, USA, 72504) and Eosin-Y (Richard-Allan Scientific, 71204) according to manufacturer’s protocols. Sections were dehydrated in increasing concentrations of ethanol, cleared in xylene, dried and coverslipped with Cytoseal Mounting Medium (Richard-Allan Scientific, 48212–187).

### Western blots and immunoprecipitation

Whole-cell lysates were obtained by applying RIPA buffer directly to adherent cells and scraping. Detached cells were pelleted from culture media and combined with the rest of the corresponding lysate. For isolation of mitochondria, cells were scraped in mitochondrial isolation buffer containing sucrose (250 mM; Sigma-Aldrich; 179949), EDTA (1 mM; Sigma-Aldrich; E4884), HEPES (10 mM; Sigma-Aldrich; H3375), PhosSTOP (Sigma-Aldrich; 4906837001) and protease inhibitors pH adjusted to 7.4. Cells were then mechanically disrupted by passage through a 27 1/2-gauge needle five times. Nuclear debris were spun out by centrifuging lysates at 15,000 x g for 10 min at 4°C and supernatants were collected.

Proteins were quantified using bicinchoninic acid solution (Sigma-Aldrich; B9643). Equal amounts of protein were run in 4 to 20% Tris-glycine SDS-PAGE gels (Life Technologies; EC6025) and transferred to nitrocellulose membranes. Membranes were blocked in 5% nonfat dry milk in Tris-buffered saline with Tween 20 (TBS-T) for 1 h at room temperature and then incubated in primary antibody diluted in 5% nonfat dry milk overnight at 4°C. Primary antibodies used were as follows: VP1 (1:1000; Vector Laboratories; VP-E603), LC3 (1:1000; Cell Signaling; 4108), TOM70 (1:1000; Proteintech; 14528-1-AP), CD63 (1:1000; System Biosciences; EXOAB-CD63A-1), PhosphoPlus TBK1/NAk (ser 172) Antibody Duet (1:1000, Cell Signaling; 61223s), Anti-Human IFNAR2 (1:500, PBL Assay Science; 21385–1), Flotillin-1 (1:1000, Santa Cruz Biotechnology; sc-74566), GABARAPL1 (1:250, Santa Cruz Biotechnology; sc-377300), GABARAPL2 (1:1000, Santa Cruz Biotechnology; 14256S), p-IRF-3 (1:1000, Cell Signaling; 29047S), APG5 (1:1000, Santa Cruz Biotechnology; sc-133158), Reticulon-3 (1:1000, Santa Cruz Biotechnology; sc-374599).

Immunoprecipitation was performed using protein A/G PLUS-Agarose beads (Santa Cruz Biotechnology; sc-2003) following manufacturer protocol.

### Plaque assays

Plaque assays were performed as previously described [27]. Briefly, HeLa cells were grown to confluency in 6 well plates. Media were removed from cells, and 400 μL serially diluted cell supernatant or pancreas sample was added on top of cells. After one hour of incubation with occasional rocking, infected cells were overlain with 4 mL 50:50 mixture of 1.2% molten agar combined with 2× DMEM. Plates were then incubated at 37 °C for 48 h and agar plugs were subsequently fixed for 20 min with 2 mL plaque fixative containing 25% acetic acid and 75% methanol. Plugs were removed and fixed cells were stained for one hour with 2.34% crystal violet solution. Cells were then washed, and plaques were counted.

### Statistical analysis

Statistical significance was determined using two-way ANOVA. Differences were measured relative to growth medium controls. Groups were considered significantly different *if p* values were less than 0.05. Error bars in figures indicate standard error.

## Supporting information

S1 Fig Intracellular and extracellular viral titers in *TBK1*^*+/+*^ and *TBK1*^*-/-*^ MEFs. *TBK1*^*+/+*^ and *TBK1*^*-/-*^ MEFs were infected with an MOI 10 for 24 h. (A) Plaque assays of intracellular viral titers were performed. ***, *p* < 0.001, Student *t* test; n = 3. (B) Plaque assays of extracellular viral titers were performed. ****, *p* < 0.0001, Student *t* test; n = 3. Data are representative of 2 experiments.
(TIF)

S2 Fig Multi-step and single-step growth curves of CVB in *siSCRAMBLE* and *siTBK1* cells. (A) A multi-step viral growth curve was performed. *siSCRAMBLE* or *siTBK1* cells were infected with an MOI 0.001 over a time course of 0 h 12 h, 24 h, and 36 h. Plaque assays of intracellular and extracellular virus were performed at the indicated time points. (B) A single-step viral growth curve was performed. Cells were infected at an MOI 5 over a time course of 0 h, 2 h, 4 h, 6 h, and 8 h. Plaque assays of intracellular or extracellular virus were performed at the indicated time points. (C) *siSCRAMBLE* or *siTBK1* cells were infected with an MOI 0.1 for 6 h. Fluorescence microscopy of HeLa cells infected at 6 h p.i. Phase contrast images show similar cell density. Scale bars represent 100 μm. (D) Cell lysates were analyzed by Western blot and densitometry quantification was performed. Student *t* test; n = 3. WL = whole lysate. Data are representative of 2 experiments.
(TIF)

S3 Fig Extracellular vesicles contain viral protein VP1 and are not derived from intracellular membranes. EVs were isolated from supernatants of cells that were mock-infected or infected with CVB for 24 h at MOI 0.1. (A) EVs were lysed in RIPA buffer and EV lysates were analyzed by Western blot for VP1, ALIX, flotillin-1, or CD63. (B) EVs were immunoprecipitated with flotillin-1 and immunoblotted for VP1, flotillin-1, and CD63. (C) WL or EVs were immunoprecipitated with reticulon-3 and immunoblotted for VP1 and reticulon-3. IP = immunoprecipitation. WL = whole lysate. Data are representative of 2 experiments.
(TIF)

S4 Fig Silencing *TBK1* increases the release of GABARAPL1 and GABARAPL2 in EVs. (A) EV lysates from *siSCRAMBLE* and *siTBK1* cells were analyzed by Western blot. Densitometric quantification of GABARAPL1 and GABARAPL2. *, *p* < 0.05, **, *p* < 0.01, ****, *p* < 0.0001, two-way ANOVA; n = 3. Data are representative of 3 experiments.
(TIF)

S5 Fig Individual silencing of *GABARAPL1* or *GABARAPL2* does not alter CVB infection. (A) Cells were treated with siRNA targeting GABARAPL1 (*siGABA1*). Cells were infected with CVB at MOI 0.01 for 24 h; n = 3. Data are representative of 2 experiments. (C) Cells were treated with siRNA targeting GABARAPL2 (*siGABA2*). Cells were infected with CVB at MOI 0.01 for 24 h; n = 3. Data are representative of 2 experiments. WL = whole lysate.
(TIF)

S6 Fig Increase in LC3-II occurs independent of productive infection. Cells were mock infected or infected with CVB, UV-inactivated (UV CVB), or heat-inactivated CVB (Heat CVB) at MOI 0.1 for 24 h. (A) Western blot of cell lysates including densitometry. (B) Western blot of EV lysates including densitometry. Data are representative of 2 experiments. WL = whole lysate.
(TIF)
