# Supplementary figures and images for: TBK1 and GABARAP family members suppress Coxsackievirus B infection by limiting viral production and promoting autophagic degradation of viral extracellular vesicles
Source: PLoS Pathog. 2022 Aug 31;18(8):e1010350. doi: 10.1371/journal.ppat.1010350 (PMC9469980; doi:10.1371/journal.ppat.1010350)

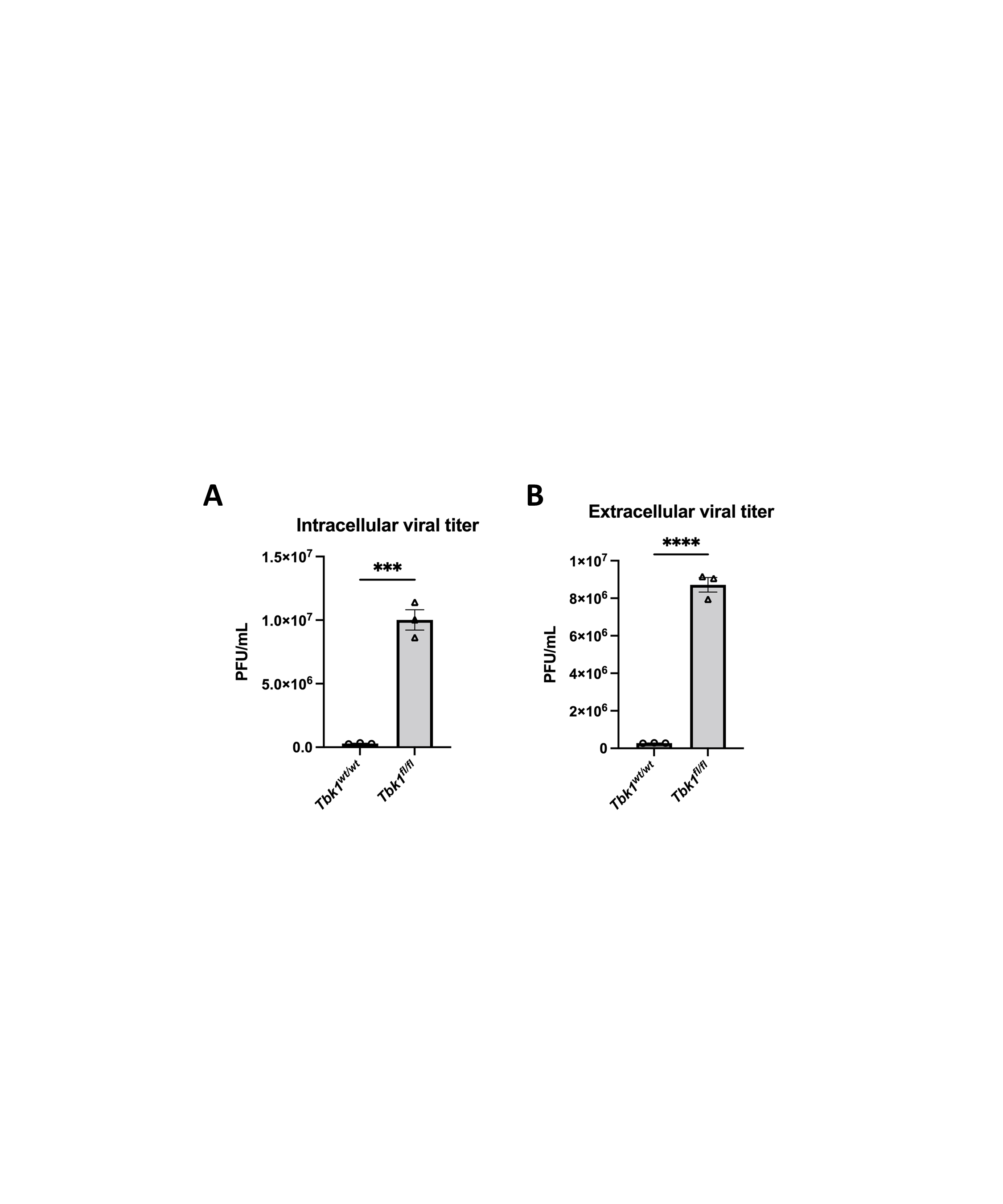

Supplement: S1 Fig — TBK1+/+ and TBK1-/- MEFs were infected with an MOI 10 for 24 h. (A) Plaque assays of intracellular viral titers were performed. ***, p < 0.001, Student t test; n = 3. (B) Plaque assays of extracellular viral titers were performed. ****, p < 0.0001, Student t test; n = 3. Data are representative of 2 experiments. (TIF) [file ppat.1010350.s001.TIF]

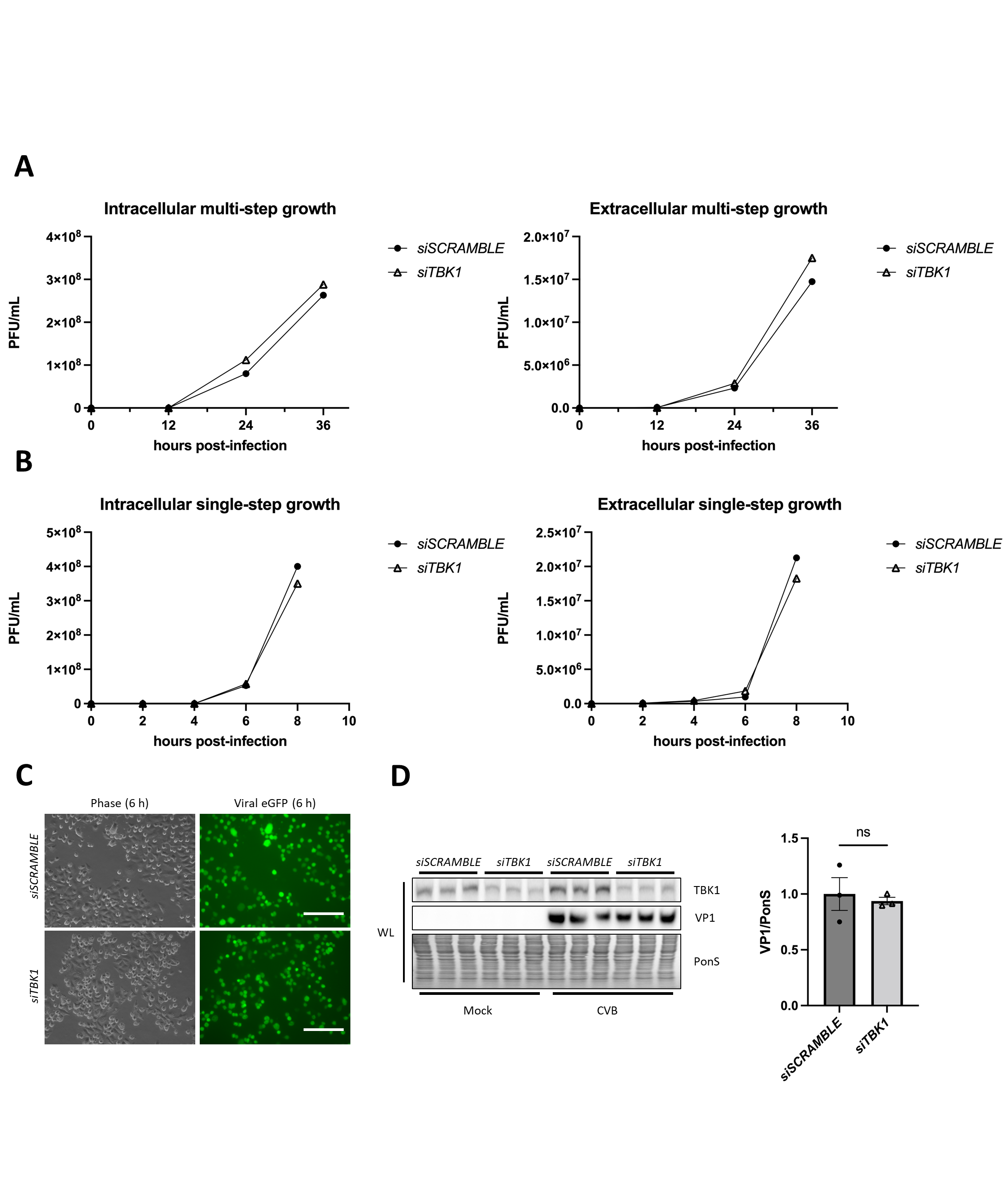

Supplement: S2 Fig — (A) A multi-step viral growth curve was performed. siSCRAMBLE or siTBK1 cells were infected with an MOI 0.001 over a time course of 0 h 12 h, 24 h, and 36 h. Plaque assays of intracellular and extracellular virus were performed at the indicated time points. (B) A single-step viral growth curve was performed. Cells were infected at an MOI 5 over a time course of 0 h, 2 h, 4 h, 6 h, and 8 h. Plaque assays of intracellular or extracellular virus were performed at the indicated time points. (C) siSCRAMBLE or siTBK1 cells were infected with an MOI 0.1 for 6 h. Fluorescence microscopy of HeLa cells infected at 6 h p.i. Phase contrast images show similar cell density. Scale bars represent 100 μm. (D) Cell lysates were analyzed by Western blot and densitometry quantification was performed. Student t test; n = 3. WL = whole lysate. Data are representative of 2 experiments. (TIF) [file ppat.1010350.s002.TIF]

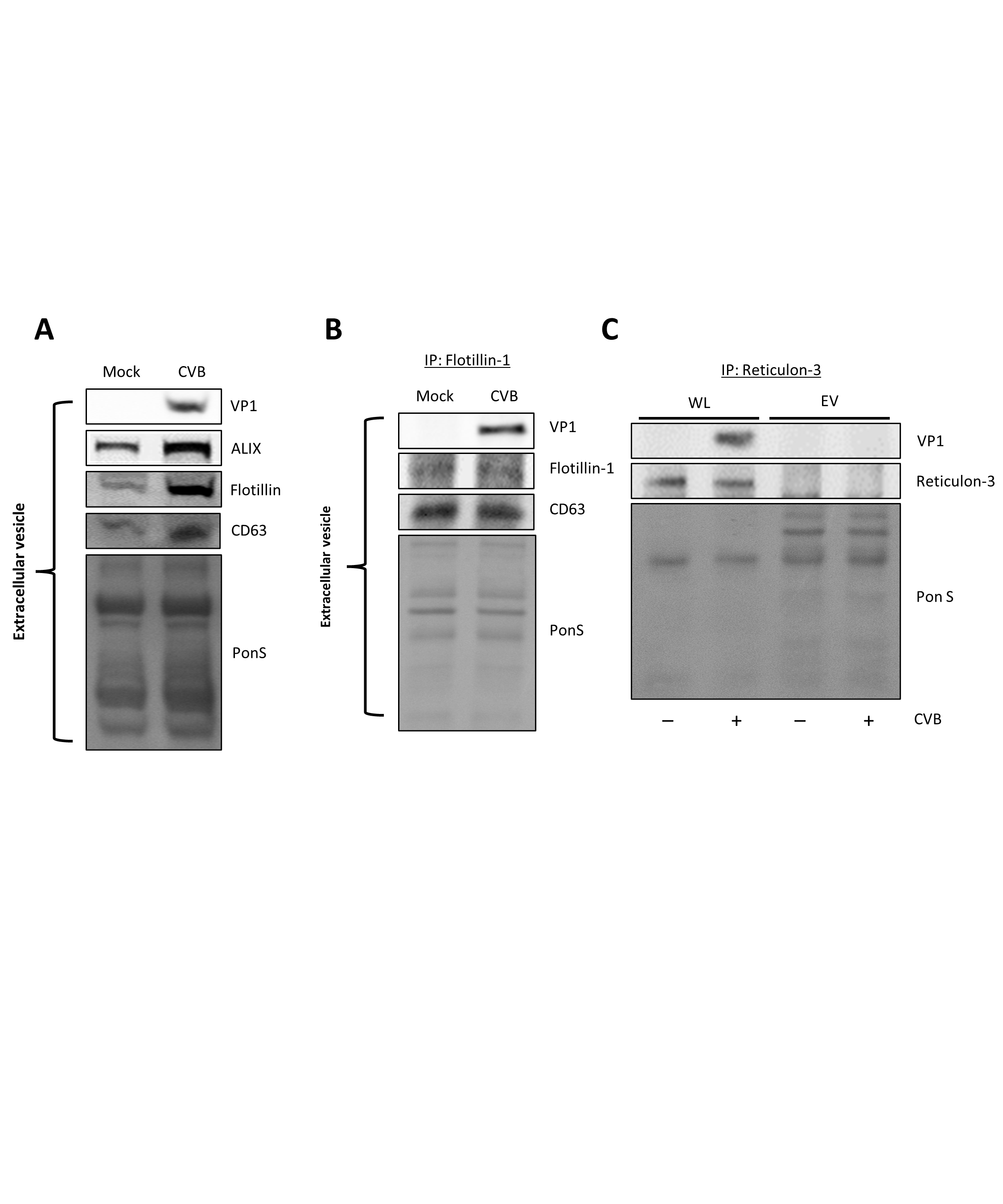

Supplement: S3 Fig — EVs were isolated from supernatants of cells that were mock-infected or infected with CVB for 24 h at MOI 0.1. (A) EVs were lysed in RIPA buffer and EV lysates were analyzed by Western blot for VP1, ALIX, flotillin-1, or CD63. (B) EVs were immunoprecipitated with flotillin-1 and immunoblotted for VP1, flotillin-1, and CD63. (C) WL or EVs were immunoprecipitated with reticulon-3 and immunoblotted for VP1 and reticulon-3. IP = immunoprecipitation. WL = whole lysate. Data are representative of 2 experiments. (TIF) [file ppat.1010350.s003.TIF]

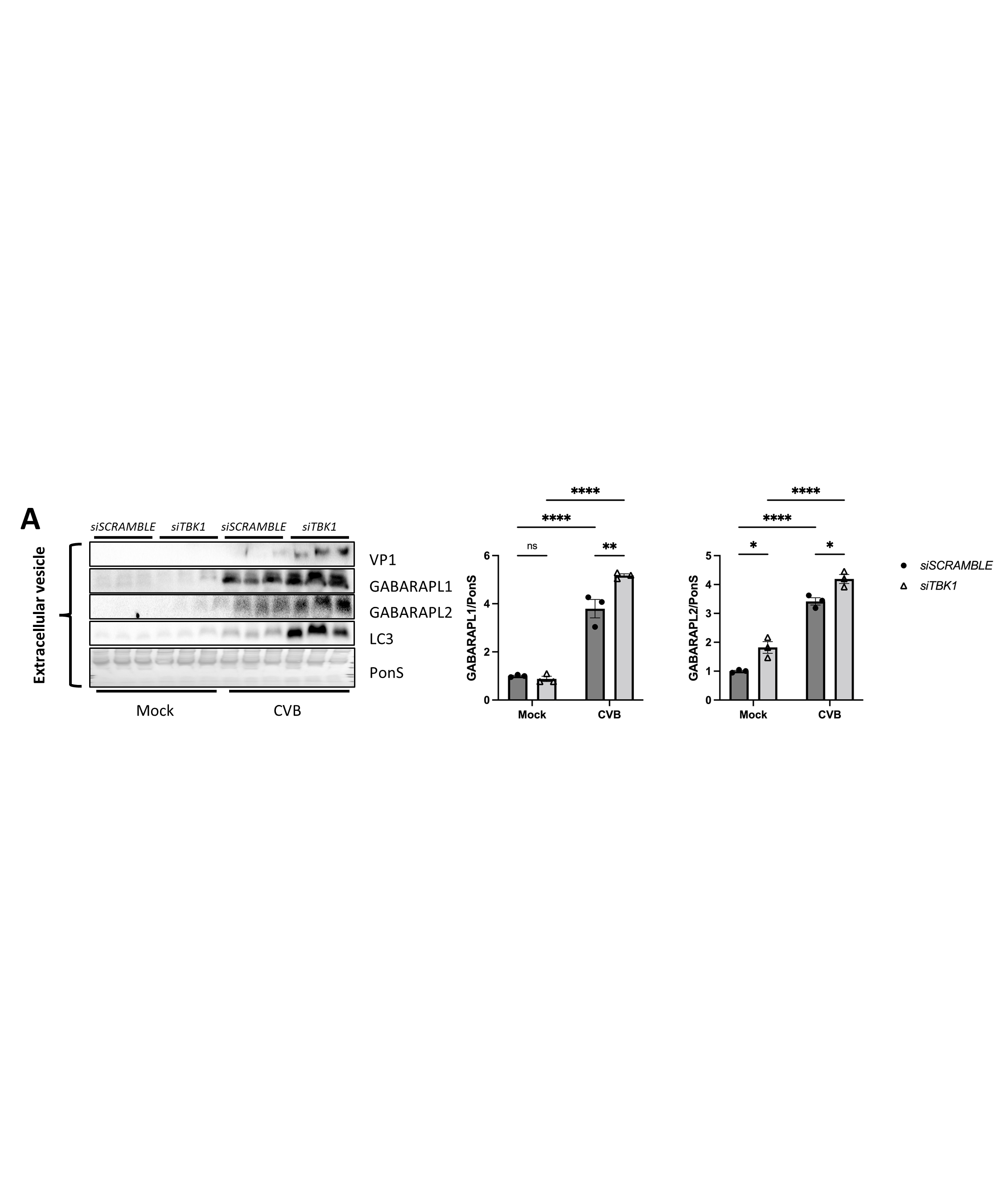

Supplement: S4 Fig — (A) EV lysates from siSCRAMBLE and siTBK1 cells were analyzed by Western blot. Densitometric quantification of GABARAPL1 and GABARAPL2. *, p < 0.05, **, p < 0.01, ****, p < 0.0001, two-way ANOVA; n = 3. Data are representative of 3 experiments. (TIF) [file ppat.1010350.s004.TIF]

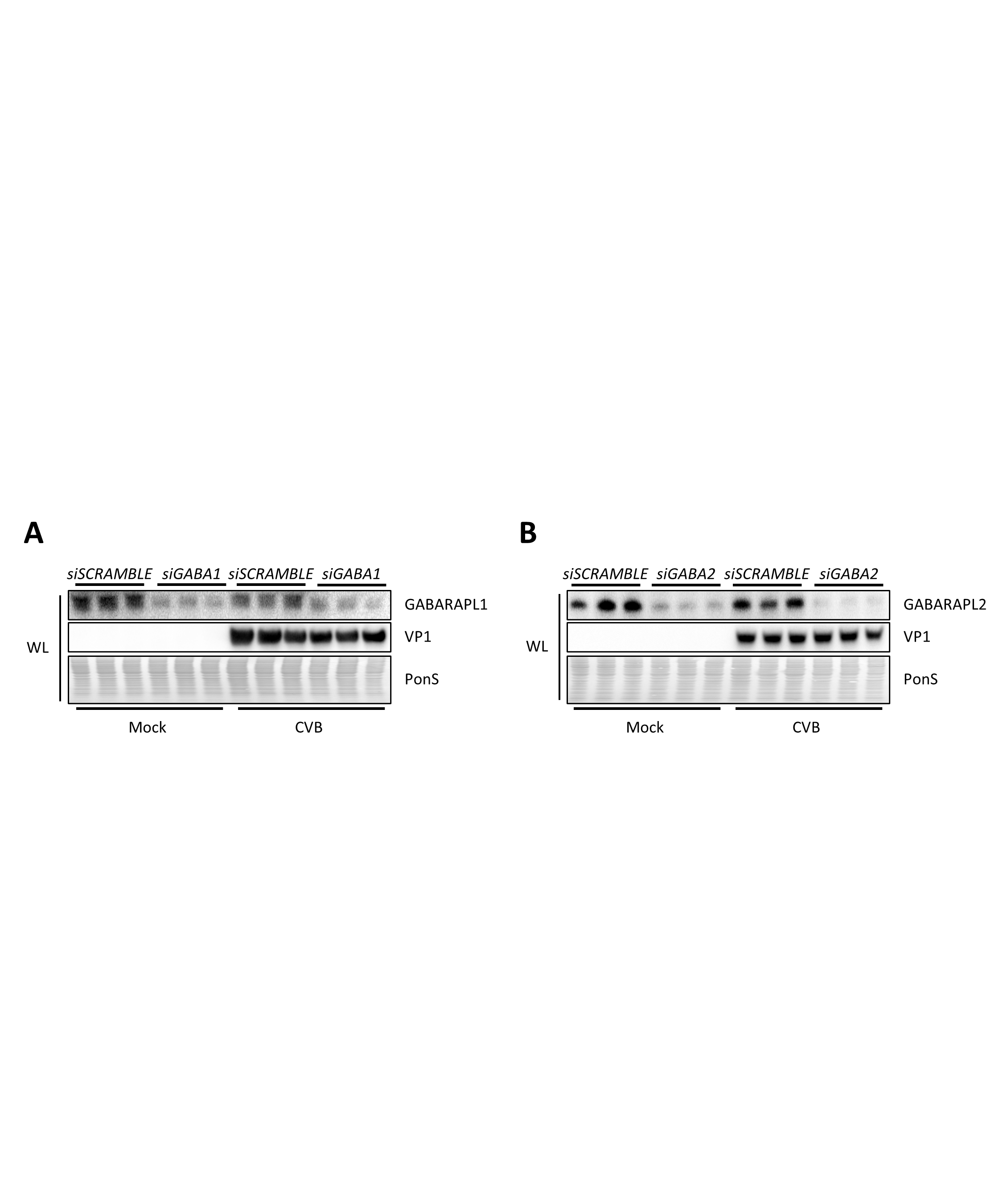

Supplement: S5 Fig — (A) Cells were treated with siRNA targeting GABARAPL1 (siGABA1). Cells were infected with CVB at MOI 0.01 for 24 h; n = 3. Data are representative of 2 experiments. (C) Cells were treated with siRNA targeting GABARAPL2 (siGABA2). Cells were infected with CVB at MOI 0.01 for 24 h; n = 3. Data are representative of 2 experiments. WL = whole lysate. (TIF) [file ppat.1010350.s005.TIF]

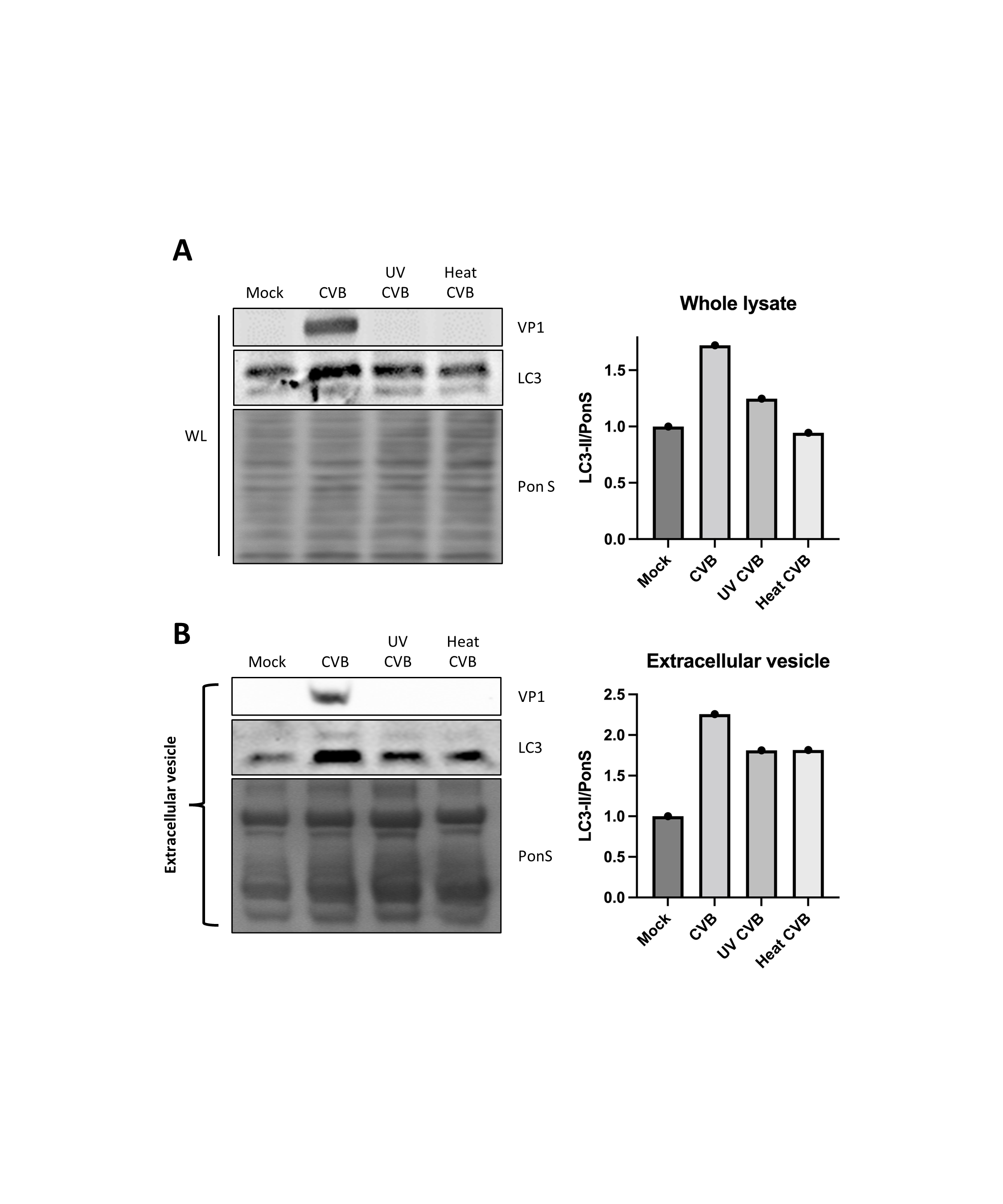

Supplement: S6 Fig — Cells were mock infected or infected with CVB, UV-inactivated (UV CVB), or heat-inactivated CVB (Heat CVB) at MOI 0.1 for 24 h. (A) Western blot of cell lysates including densitometry. (B) Western blot of EV lysates including densitometry. Data are representative of 2 experiments. WL = whole lysate. (TIF) [file ppat.1010350.s006.TIF]
